# mTORC1 controls murine postprandial hepatic glycogen synthesis via *Ppp1r3b*

**DOI:** 10.1172/JCI173782

**Published:** 2024-01-30

**Authors:** Kahealani Uehara, Won Dong Lee, Megan Stefkovich, Dipsikha Biswas, Dominic Santoleri, Anna Garcia Whitlock, William Quinn, Talia Coopersmith, Kate Townsend Creasy, Daniel J. Rader, Kei Sakamoto, Joshua D. Rabinowitz, Paul M. Titchenell

**Affiliations:** 1 Institute for Diabetes, Obesity, and Metabolism,; 2 Biochemistry and Molecular Biophysics Graduate Group, and; 3 Department of Physiology, Perelman School of Medicine, University of Pennsylvania, Philadelphia, Pennsylvania, USA.; 4 Lewis Sigler Institute for Integrative Genomics,; 5 Department of Chemistry, and; 6 Ludwig Institute for Cancer Research, Princeton Branch, Princeton, New Jersey, USA.; 7 Novo Nordisk Foundation Center for Basic Metabolic Research, University of Copenhagen, Copenhagen, Denmark.; 8 Department of Surgery,; 9 Department of Medicine, Division of Translational Medicine and Human Genetics, and; 10 Department of Biobehavioral Health Sciences, School of Nursing, University of Pennsylvania, Philadelphia, Pennsylvania, USA.; 11 Department of Molecular Biology, Princeton University, Princeton, New Jersey, USA.

**Keywords:** Endocrinology, Metabolism, Glucose metabolism, Insulin signaling

## Abstract

In response to a meal, insulin drives hepatic glycogen synthesis to help regulate systemic glucose homeostasis. The mechanistic target of rapamycin complex 1 (mTORC1) is a well-established insulin target and contributes to the postprandial control of liver lipid metabolism, autophagy, and protein synthesis. However, its role in hepatic glucose metabolism is less understood. Here, we used metabolomics, isotope tracing, and mouse genetics to define a role for liver mTORC1 signaling in the control of postprandial glycolytic intermediates and glycogen deposition. We show that mTORC1 is required for glycogen synthase activity and glycogenesis. Mechanistically, hepatic mTORC1 activity promotes the feeding-dependent induction of *Ppp1r3b*, a gene encoding a phosphatase important for glycogen synthase activity whose polymorphisms are linked to human diabetes. Reexpression of *Ppp1r3b* in livers lacking mTORC1 signaling enhances glycogen synthase activity and restores postprandial glycogen content. mTORC1-dependent transcriptional control of *Ppp1r3b* is facilitated by FOXO1, a well characterized transcriptional regulator involved in the hepatic response to nutrient intake. Collectively, we identify a role for mTORC1 signaling in the transcriptional regulation of *Ppp1r3b* and the subsequent induction of postprandial hepatic glycogen synthesis.

## Abstract 2

A signaling pathway connects mTORC1 to glycogen synthase to control liver glycogen content via PPP1R3B, a protein phosphatase 1 subunit essential for hepatic glycogen synthesis.

## Graphical abstract

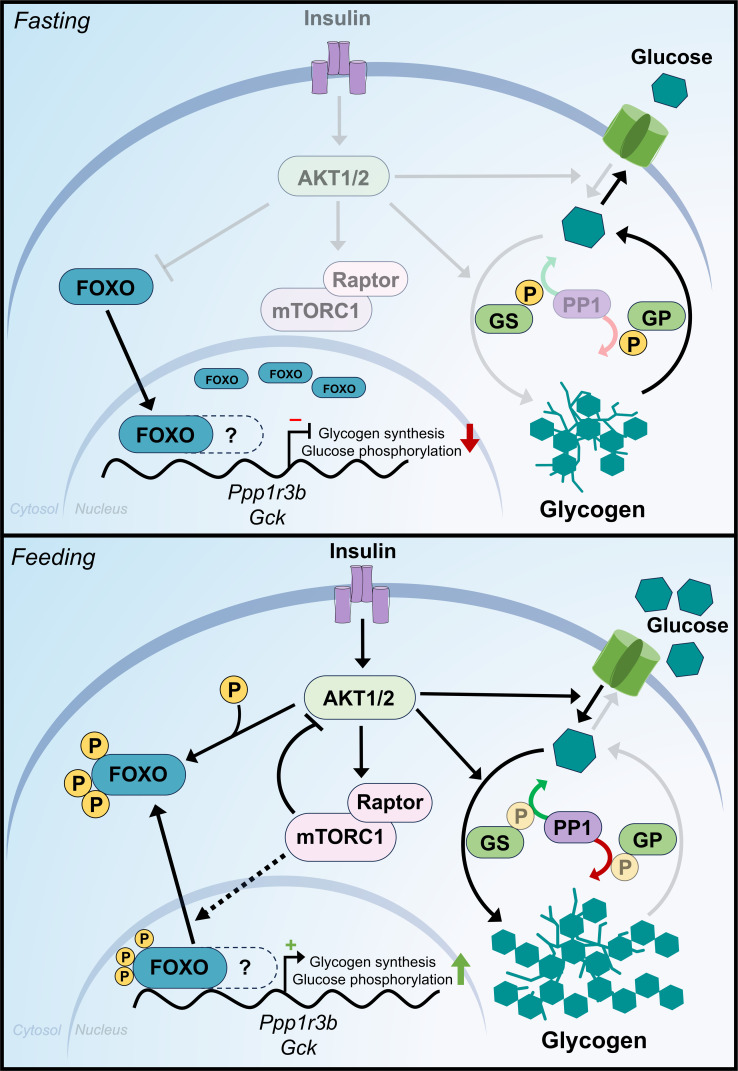

## Introduction

The liver is a central regulator of systemic glucose metabolism. Dysregulation of postprandial hepatic glucose metabolism contributes to the development of metabolic disorders, such as insulin resistance and type II diabetes (1). During periods of fasting, the liver breaks down glycogen stores via glycogenolysis to produce glucose and maintain circulating blood glucose levels (2, 3). Upon feeding, blood glucose rises, causing an increase in insulin secretion from the pancreas, which drives anabolic metabolism in insulin-responsive tissues such as the liver, skeletal muscle, and adipose tissue.

In the postprandial liver, glucose is taken up and stored as glycogen, through a process called glycogenesis. Glycogen is synthesized from 2 pathways: (a) the direct pathway via glucose phosphorylation via glucokinase (GCK), the main hexokinase of the liver, and (b) the indirect pathway, in which gluconeogenic substrates fuel the generation of glycogen precursors. Overall, glycogenesis is driven by glucose-6-phosphate (G6P) and its conversion to glucose-1-phosphate (G1P) by phosphoglucomutase (4, 5). G1P then reacts with uridine triphosphate (UTP) to generate uridine diphosphate glucose (UDP-glucose) via UDP-glucose pyrophosphorylase (UGP) (6). UDP-glucose is transferred to glycogen branches via glycogen synthase (GS), contributing to glycogen stores. A feedforward mechanism exists whereby G6P allosterically activates GS to stimulate glycogenesis (7–9). Additionally, phosphorylation of GS via glycogen synthase kinase 3 (GSK3) inhibits GS enzymatic activity and its dephosphorylation via protein phosphatase 1 (PP1) leads to GS activation (10, 11). Glycogen storage is antagonized by glycogen phosphorylase (GP), the enzyme required for the phosphorylation of glycogen branches to generate and release G1P (12, 13). PP1-mediated dephosphorylation of GP renders the enzyme inactive (10). Defects in the key enzymes involved in glycogen synthesis and breakdown underpin a variety of glycogen storage diseases (GSD) (14). Furthermore, deficiency in the enzymes regulating GS and GP activities, namely PP1, associate with abnormal glycogen content (15).

PP1 regulatory subunit 3B (PPP1R3B) is a feeding-induced, glycogen-targeting subunit of PP1 required for GS dephosphorylation and thus glycogenesis in the liver (16–19). Whole-body and liver-specific deletion of *Ppp1r3b* in mice prevents postprandial hepatic glycogen storage (16, 17). Hepatic overexpression of *Ppp1r3b* in mice enhances glycogen storage, highlighting the importance of *Ppp1r3b* expression for glycogen maintenance (16, 17, 19). Notably, the meta-analyses of glucose- and insulin-related traits consortium (MAGIC) identified variants near the *PPP1R3B* locus that are associated with fasting insulin and fasting glucose (20, 21). Given its importance in regulating postprandial glycogen storage and that genetic variations of *PPP1R3B* are associated with glycemic traits, *PPP1R3B* remains a gene of interest for type 2 diabetes (T2D). However, the upstream signals controlling the regulation of *PPP1R3B* remain ill defined.

Hepatic insulin action is critical for postprandial glycogen synthesis and the suppression of glucose production. Under conditions of insulin resistance, insulin fails to stimulate postprandial glycogen synthesis and suppress glucose production, contributing to hyperglycemia (22). Insulin acts through the PI3K/AKT axis to stimulate glucose uptake and inhibit hepatic glucose production. Insulin action through AKT is required for stimulation of glycogenesis and inhibition of gluconeogenesis, through which GSK3 and forkhead box O (FOXO) transcription factors are involved, respectively. Interestingly, although downstream of AKT, the mechanistic target of rapamycin complex 1 (mTORC1) has not been previously demonstrated to contribute to hepatic glucose control. Several published reports show an important role for mTORC1 in liver biology including effects on protein synthesis, cell growth, and lipid synthesis and secretion (23). However, there is a dearth of information for mTORC1’s involvement in liver glucose metabolism. Long-term treatment of rapamycin in canines with glycogen storage disease IIIa (GSDIIIa), an autosomal disorder caused by a defect in glycogen debranching enzyme, reduces liver glycogen levels (24). In a separate study using immortalized hepatocytes (HepG2), insulin-mediated GS activation is blunted in response to rapamycin treatment (25). On the other hand, activation of mTORC1 via deletion of tuberous sclerosis 2 (TSC2), a negative regulator of mTORC1, caused an increase in intracellular glycogen content in MEFs (26). These data suggest that mTORC1 may promote glycogen storage; however, the underlying mechanisms are not known.

In this study, we demonstrate a requirement for hepatic mTORC1 signaling on glycolytic intermediates and postprandial hepatic glucose disposal via mTORC1-dependent control of GS activity. First, we performed metabolomics in an acute liver-specific model of mTORC1 inhibition (referred to here as L-Raptor-KO) to identify changes in glycolytic metabolites dependent on mTORC1. Using ^13^C-glucose tracing, we found that mTORC1 activity was required for feeding-induced glycogen synthesis. Mechanistically, we show that mTORC1 was required for the postprandial induction of *Ppp1r3b* mRNA expression and demonstrate that reexpression of *Ppp1r3b* in an mTORC1-null liver is sufficient to restore GS activity and feeding-induced hepatic glycogen synthesis. The mTORC1-dependent transcriptional regulation of *Ppp1r3b* is due, in part, to FOXO retention in the nucleus, rendering constitutive repression of *Ppp1r3b* and *Gck* transcripts. Collectively, data presented here describe a mechanism for mTORC1 in the control of postprandial hepatic glucose storage and GS activity.

## Results

### Postprandial metabolomics reveal increased glycogen precursors in the absence of mTORC1 activity.

**Figure 1:**
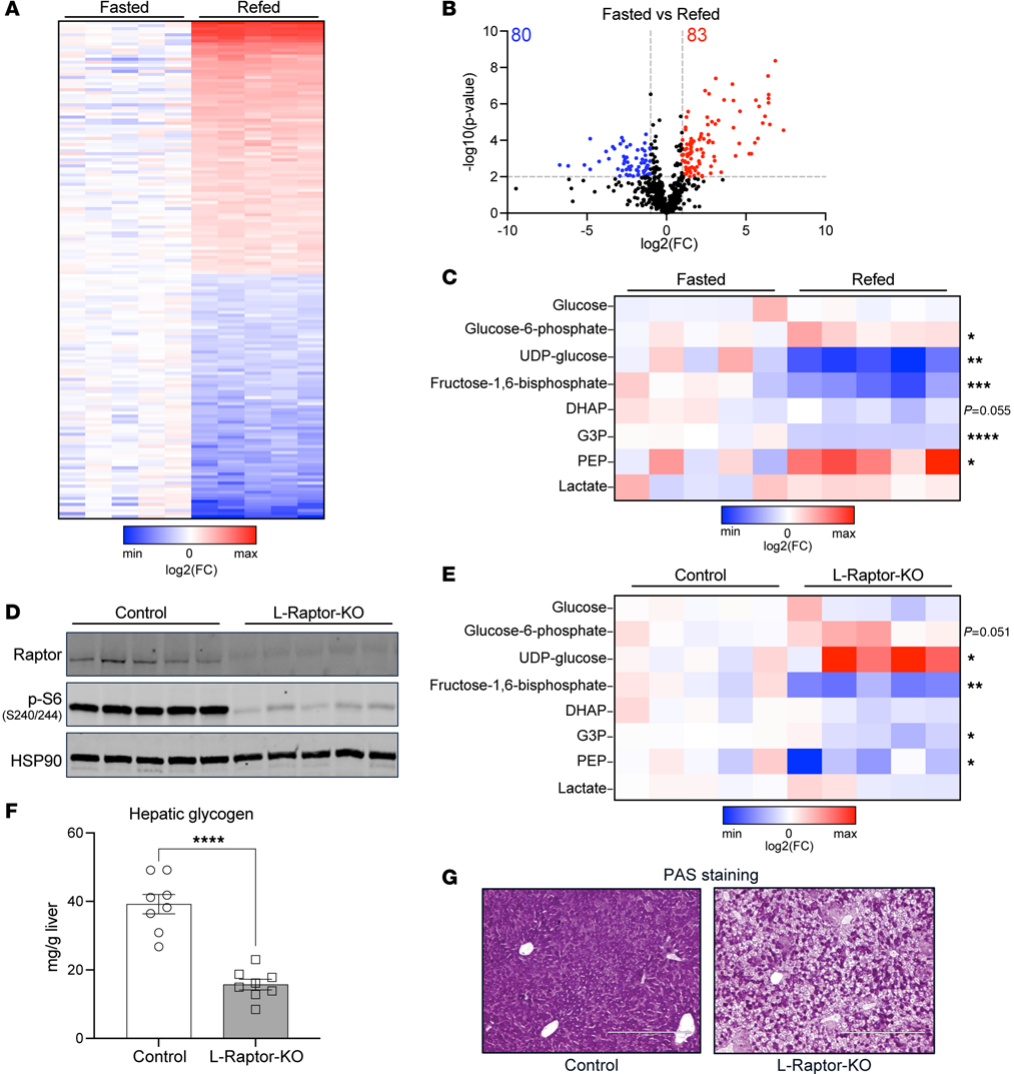
Postprandial metabolomics reveal increased glycogen precursors in the absence of mTORC1 activity. (**A**–**C**) Mice aged 10 to 12 weeks were fasted for 16 hours (Fasted) then given food for 4 hours (Refed). (**A**) Heat map of differential metabolite abundance shown as log_2_ (FC) compared with fasted livers. (**B**) Volcano plot showing –log_10_(*P* value versus fasted) on y-axis and log_2_ (FC versus fasted) on x-axis. Blue dots represent log_2_ (FC) < –2, *P* < 0.01. Red dots represent llog_2_ (FC) > 2, and *P* < 0.01. (**C**) The relative abundance of selected glucose metabolites. (**D**–**G**) *Rptor*^loxP/loxP^ mice aged 10–12 weeks were injected with AAV8-TBG-Cre (L-Raptor-KO) or AAV8-TBG-GFP (Control). Two weeks after injection, mice were fasted overnight, then chow was reintroduced for 4 hours before sacrifice. (**D**) Immunoblot demonstrating loss of Raptor protein and inhibition of mTORC1 signaling. (**E**) Heat map of selected glucose metabolite relative abundance shown as log_2_ (FC) compared with control fed livers. (**F**) Hepatic glycogen in fed livers. Data shown as mean ± SEM. (**G**) PAS staining for glycogen (pink). Scale bar: 400 μm. *P < 0.05, **P < 0.01, ***P < 0.001, ****P < 0.0001 versus WT fasted via student’s *t* test. Red indicates higher metabolite abundance.

In response to feeding and elevated systemic insulin levels, the liver rapidly shifts from a state of catabolism to anabolism. Our lab and others have focused on defining the global transcriptional response to nutrient intake (27). However, the acute changes in liver metabolites that occur with feeding are less defined. To understand how the liver metabolome changes in response to feeding,, livers from mice in both a fasting and prandial state were subjected to metabolomic analysis(Figure 1, A and B). Of the 739 metabolites identified, 163 metabolites were differentially regulated (FC greater than 2, or FC less than –2, *P* < 0.01) (Figure 1, A and B). With respect to glucose metabolism, while G6P and phosphoenolpyruvate (PEP) increased, fructose-1,6-bisphosphate (FBP), glycerol-3-phosphate (G3P), and UDP-glucose decreased, with the strongest change in UDP-glucose (Figure 1C). G6P is analytically indistinguishable from other isomers in the liquid chromatography methods used, but it is the most abundant hexose phosphate, hence, we refer to this hexose phosphate as G6P.

Given that mTORC1 is a critical feeding-regulated kinase in hepatocytes (23), we generated mice lacking mTORC1 specially in hepatocytes from adult mice, to define mTORC1’s role in the postprandial response. To do so, *Rptor*^loxP/loxP^ mice were injected with a liver-specific adeno-associated virus (AAV), serotype 8, carrying GFP (Control) or Cre recombinase (L-Raptor-KO). Raptor is an essential subunit of the mTORC1 complex and deletion leads to complete loss of mTORC1 activity without affecting mTORC2 (28). Two weeks after AAV injections, Raptor mRNA (Supplemental Figure 1A; supplemental material available online with this article; https://doi.org/10.1172/JCI173782DS1) and protein (Figure 1D) levels were reduced in L-Raptor-KO, and phosphorylation of the canonical mTORC1 downstream target ribosomal protein S6 decreased (Figure 1D). Metabolomic analysis of the postprandial livers from control and L-Raptor-KO mice revealed that, out of the total 739 metabolites screened, 136 metabolites were upregulated (FC greater than 2) in the absence of mTORC1 signaling (Supplemental Figure 1, B and C). Notably, a general decrease in glycolysis-related metabolites downstream of G6P, including fructose 1,6-bisphosphate, glycerol 3-phosphate and PEP, was noted (Figure 1E). Surprisingly, there was an increase in the more proximal glycolytic metabolites, including the glycogen precursors, UDP-glucose, and G6P (*P* = 0.051) (Figure 1E).

An explanation for the increased levels of postprandial UDP-glucose observed in L-Raptor-KO mice could be impaired consumption by glycogen synthesis. Accordingly, the levels of postprandial glycogen content were determined following loss of mTORC1 signaling. Postprandial liver glycogen content was significantly decreased in mTORC1-deficient livers, evidenced by both an enzymatic assay and Periodic Acid Schiff (PAS) staining (Figure 1, F and G). The reduced glycogen content in L-Raptor-KO livers was consistent with lower liver wet weights (Supplemental Figure 1D). Taken together, these data indicate that liver mTORC1 signaling is required for proper liver glycogen storage in the postprandial state.

### mTORC1 is required for hepatic glycogen synthesis.

**Figure 2:**
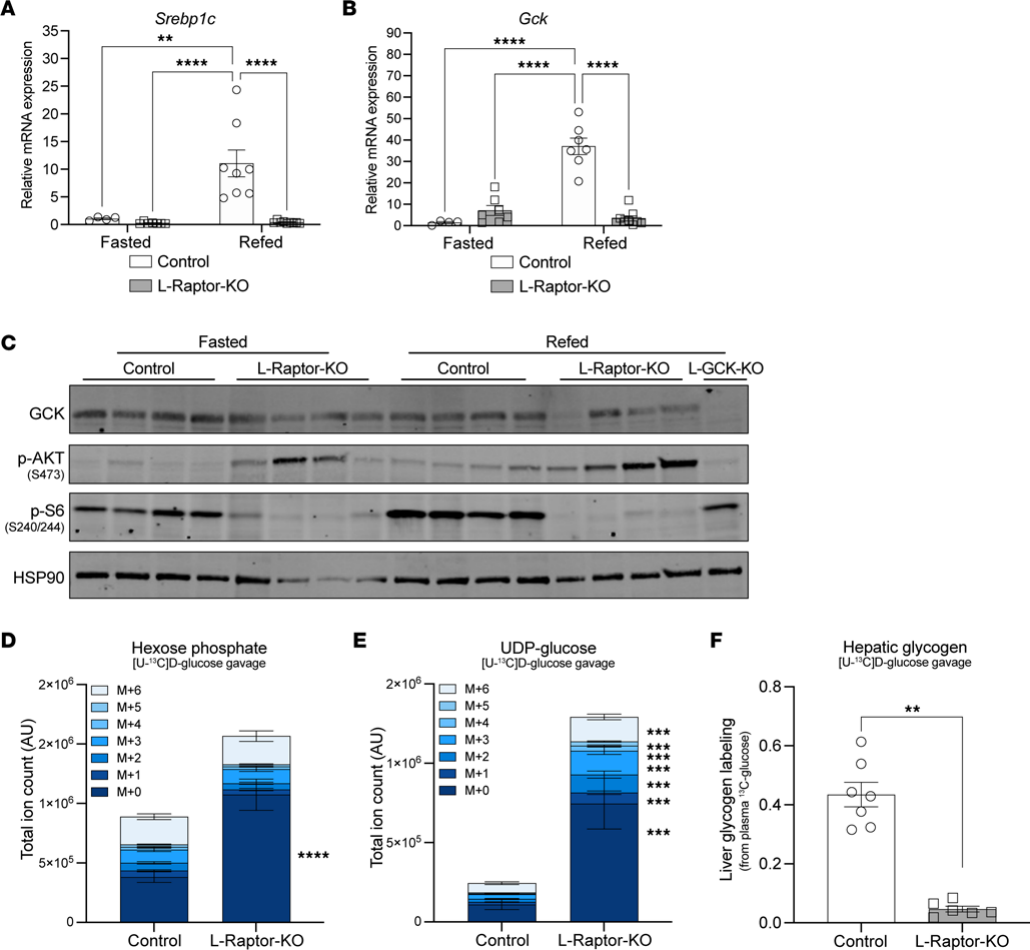
mTORC1 is required for hepatic glycogen synthesis. (**A**) *Rptor*^loxP/loxP^ or *Gck* ^loxP/loxP^ mice aged 10–12 weeks were injected with AAV-TBG-GFP (Control) or AAV-TBG-Cre (L-Raptor-KO or L-GCK-KO). (**A** and **B**) Two weeks after AAV injection, mice were fasted overnight and refed for 4 hours before sacrifice. (**A** and **B**) Gene expression of *Srebp1c* and *Gck* (glucokinase). (**C**) Immunoblot of GCK protein, activation of AKT, and inhibition of mTORC1 signaling. (**D**–**F**) Two weeks after AAV injection, mice were fasted overnight and subjected to oral gavage with 2 g/kg U-^13^C-D-glucose. Mice were sacrificed and livers were harvested 30 minutes after oral gavage. (**D** and **E**) Total ion count of hexose phosphate and UDP-glucose and respective mass isotopomer distribution in liver tissue. (**F**) Hepatic glycogen labeling representing the average carbon labeling enrichment of glycogen from oral gavage of [U-^13^C]-glucose normalized to plasma glucose labeling (Supplemental Figure 2, A and B). **P < 0.01 versus control mice, ****P < 0.0001 versus control fed mice via 2-way ANOVA (**A** and **B**) or students *t* test (**D**–**F**). Data show in ± SEM.

Based on the increased G6P and UDP-glucose levels in L-Raptor-KO livers, as well as decreased hepatic glycogen (Figure 1, E and F), we next investigated whether this was due to increased direct contribution of glucose to the G6P and UDP-glucose pools. mTORC1 signaling is required, but not sufficient, for lipogenic gene induction through activation of the transcription factor sterol regulatory binding protein 1c (SREBP1c) (29, 30). One of the downstream targets of SREBP1c is GCK, the main hexokinase and glucose sensor in the liver (5). Previous studies have highlighted the importance for insulin signaling and SREBP1c processing in *Gck* mRNA regulation (Figure 2A) (31, 32). Indeed, in mTORC1-deficient livers, a loss of mRNA expression of *Gck* in response to feeding was observed (Figure 2B). Despite this reduction in mRNA, there is only a modest reduction in GCK protein in L-Raptor-KO livers, suggesting that there is a distinction between mRNA regulation and protein regulation of GCK by mTORC1 (Figure 2C).

Next, ^13^C-glucose labeling was performed to determine how mTORC1 controlled postprandial glycogen accumulation. Overnight fasted control and L-Raptor-KO mice were administered an oral gavage with 2 g/kg [U-^13^C] glucose and the labeling of glycolytic metabolites and glycogen 30 minutes after gavage was determined. Consistent with the findings from the steady-state metabolomics, L-Raptor-KO livers contained increased pool sizes of hexose phosphate and UDP-glucose (Figure 2, D and E and Figure 1E), independent of changes in plasma glucose labeling differences (Supplemental Figure 2, A and B). However, there were no differences in isotope labeling of hexose phosphate, including M+6, implying that GCK protein is still functional in the absence of mTORC1 signaling (Figure 2, C and D and Supplemental Figure 2C). Interestingly, an increase in the pool size of isotopomers (M+1…M+6) of UDP-glucose was detected in L-Raptor-KO livers, consistent with buildup of glucose-derived carbons in UDP-glucose in L-Raptor-KO mice (Figure 2E and Supplemental Figure 2D). Consistent with impaired use of UDP-glucose to make glycogen, the enrichment of liver glycogen from circulating ^13^C-glucose was significantly reduced in L-Raptor-KO livers (Figure 2F). Altogether, these data demonstrate that mTORC1 was required for hepatic glycogen synthesis from glucose, and this was independent of alterations in GCK activity.

### mTORC1 controls glycogenesis through regulation of GS activity.

**Figure 3:**
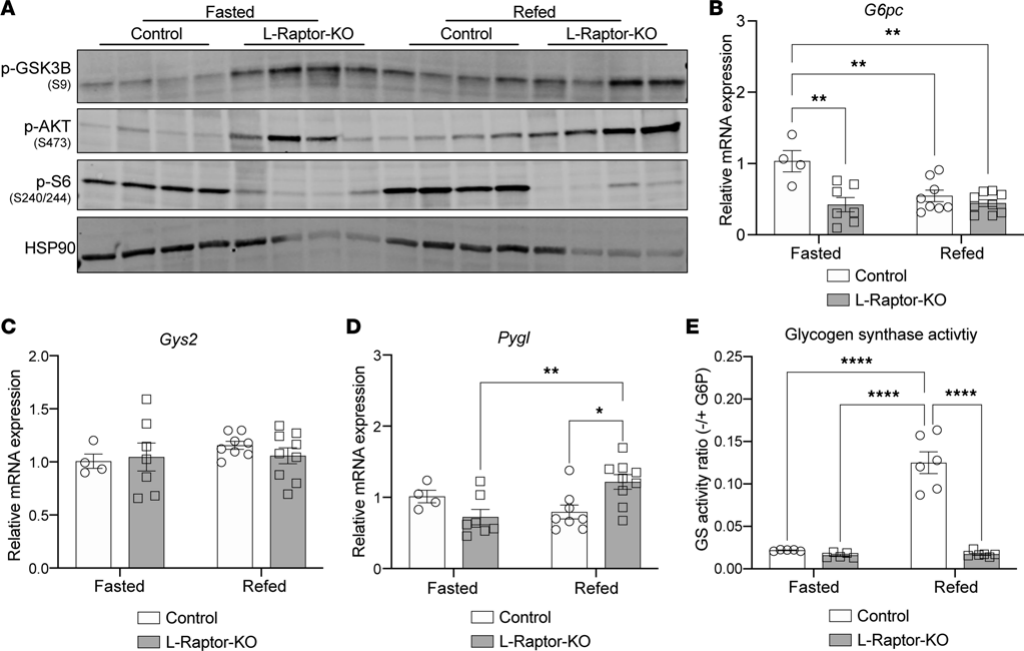
mTORC1 controls glycogenesis through regulation of GS activity. (**A**–**E**) *Rptor*^loxP/loxP^ mice aged 10–12 weeks were injected with AAV8-TBG-Cre (L-Raptor-KO) or AAV8-TBG-GFP (control). Two weeks after injection, mice were fasted overnight (fasted), or reintroduced to food for 4 hours (refed) before sacrifice. (**A**) Immunoblot of lysates from refed livers. (**B**–**D**) Relative mRNA expression of *G6pc* (glucose-6-phosphatase), *Gys2* (glycogen synthase)*,* and *Pygl* (glycogen phosphorylase), respectively. (**E**) GS activity measured as a ratio in the presence or absence of saturated G6P. **P < 0.01, ***P < 0.001, ****P < 0.0001, via 2-way ANOVA. Data shown in ± SEM.

Phosphorylation of GS renders the enzyme inactive, whereas dephosphorylation activates GS and promotes glycogenesis. GSK3 phosphorylates and negatively regulates GS. Canonically, insulin stimulates the phosphorylation of GSK3 to inhibit its catalytic function, thereby preventing GS phosphorylation and inhibition, thus promoting glycogen synthesis. In L-Raptor-KO livers, GSK3 phosphorylation levels were increased in the postprandial state, which suggests that GSK3 was likely not involved downstream of mTORC1 regulating glycogenesis (Figure 3A). These findings are associated with increased phospho-AKT signal in L-Raptor KO mice due to relief of negative feedback inhibition by mTORC1 to proximal insulin signaling (Figure 2C). In addition, the levels of mRNA expression of *G6pc*, the phosphatase that converts G6P to glucose, and *Gys2*, the gene encoding the liver GS isoform, were no different between control and L-Raptor-KO mice (Figure 3, B and C). Modest increases in *Pygl,* the gene encoding the liver GP isoform, were observed (Figure 3D). We measured GS activity and observed a significant blunting in the feeding induction of GS in the L-Raptor-KO livers compared with control animals (Figure 3E). These data suggest that mTORC1 controls postprandial hepatic glycogen synthesis in part via the regulation of GS activity, independent of increased G6P levels, a well-defined allosteric activator of GS (8).

### Restoration of Ppp1r3b in L-Raptor-KO livers promotes GS activity and glycogen storage.

**Figure 4:**
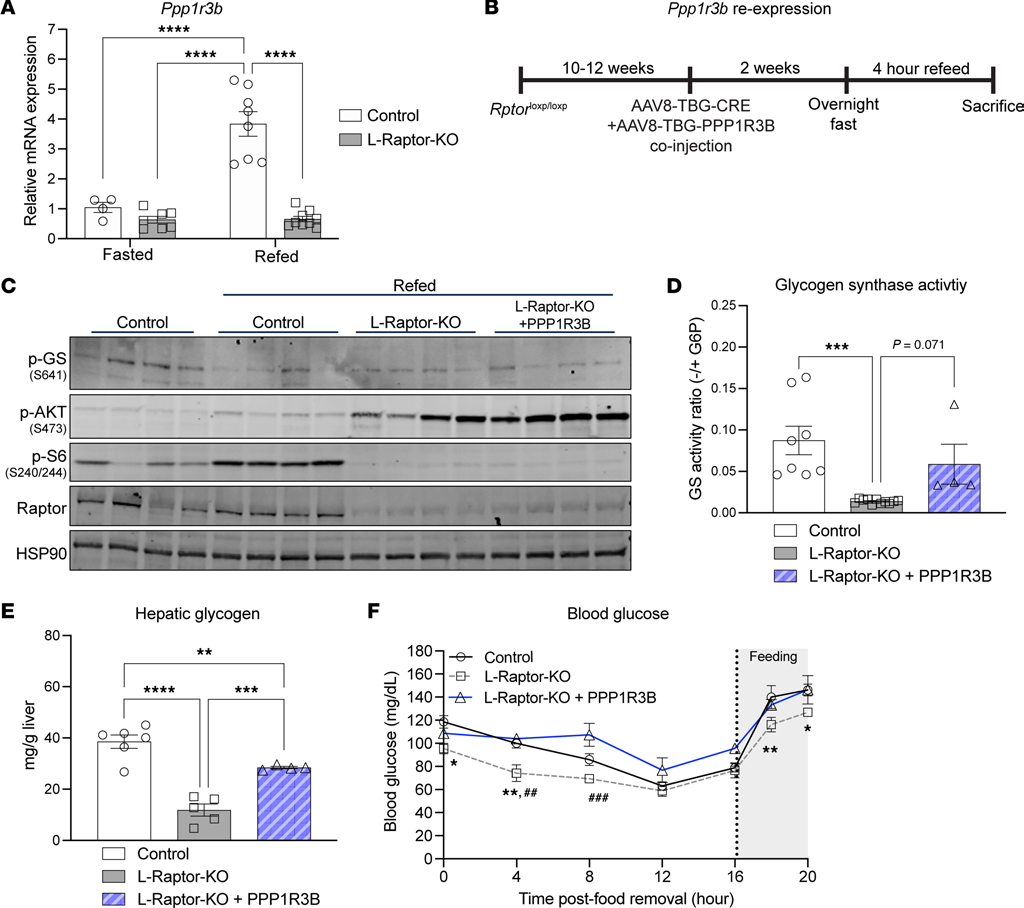
Restoration of Ppp1r3b in L-Raptor-KO livers promotes GS activity and glycogen storage. *Rptor*^loxP/loxP^ mice aged 10–12 weeks were injected with AAV8-TBG-GFP (control), AAV8-TBG-Cre in combination with AAV8-TBG-GFP (L-Raptor-KO), or AAV8-TBG-Cre in combination with AAV8-TBG-Ppp1r3b (L-Raptor-KO + Ppp1r3b), 2 weeks prior to an overnight fast and 4 hour period where food was reintroduced (refed). (**A**) Relative mRNA expression of *Ppp1r3b*. (**B**) Experimental schematic. (**C**) Immunoblot of liver lysate, indicating inhibition of mTORC1 signaling following coinjections of AAV, and changes in phosphorylation of GS. (**D**) GS activity measured as a ratio in the presence or absence of saturated G6P in refed livers. (**E**) Hepatic glycogen measured in fed livers. (**F**) Blood glucose measurement at indicated time following food removal. At hour 16, mice were given food, as indicated by “feeding” notation in the gray area**.** ##, n=5,5,3 (Control, L-Raptor-KO, L-Raptor-KO + PPP1R3B, respectively. *P < 0.05, **P < 0.01, ***P < 0.001, ****P < 0.0001 versus indicated genotype via 2-way ANOVA (**A**) or 1-way ANOVA (**D**–**F**). Data shown in ± SEM.

As mentioned previously, GS activity is regulated by phosphorylation, of which PP1 family of phosphatases are an essential component in modulating glycogen levels. PPP1R3B, also known as G_L_, is an essential regulatory subunit of PP1 complex, and genetic variations near the *PPP1R3B* locus are associated with fasting glucose and insulin (20). *Ppp1r3b* mRNA is induced upon feeding (17), and we find that this upregulation is dependent upon hepatic mTORC1 signaling (Figure 4A). These data suggest that mTORC1 regulates postprandial glycogen deposition via *Ppp1r3b* expression.

To test the sufficiency of *Ppp1r3b* in mediating the effects of mTORC1 on hepatic glycogen content, mice were coinjected with AAV8-TBG-Ppp1r3b along with AAV8-TBG-Cre, to generate a mouse reexpressing *Ppp1r3b* in Raptor-deficient hepatocytes (L-Raptor-KO + Ppp1r3b) (Figure 4B). Coinjection of AAV-Ppp1r3b and AAV-Cre resulted in deletion of the *Rptor* gene and a functional decrease in mTORC1 signaling (Supplemental Figure 3A). This coinjection strategy led to increased *Ppp1r3b* mRNA levels; albeit not to the same extent as in control mice (Supplemental Figure 3A). The partial reexpression of *Ppp1r3b* in L-Raptor-KO resulted in a modest decrease in phosphorylation of GS (p-GS) at Serine-641 compared with L-Raptor-KO alone, suggesting an increase in GS activity and glycogen synthesis (Figure 4C). This degree of an effect of AAV-PPP1R3B reexpression on Serine-641 was similar to changes reported previously (16). Notably, *PPP1r3b* reexpression correlated with an increase in GS activity (approximately 2-fold compared with L-Raptor-KO) that was indistinguishable from control mice (Figure 4D). Moreover, PPP1R3B expression increased hepatic glycogen levels significantly in the mice lacking hepatic mTORC1 signaling (Figure 4E). Physiologically, the changes in glycogen content influenced systemic glycemia as food removal induced a hypoglycemic state within 4 hours in L-Raptor-KO mice, which was completely normalized by *Ppp1r3b* reexpression (Figure 4F). Of note, expression of *Ppp1r3b* did not alter mRNA expression of *Gck* and *G6pc* (Supplemental Figure 3B). Overall, restoring *Ppp1r3b* in mTORC1-ablated livers improved postprandial GS activity and enhanced hepatic glycogen storage leading to maintenance of fasting glycemia.

### Exogenous SREBP1c expression fails to restore hepatic glycogen in L-Raptor-KO mice.

To determine which transcription factors may mediate *Ppp1r3b* expression downstream of mTORC1, the canonical fasting/feeding transcription factors were profiled. Carbohydrate responsive element-binding protein (ChREBP) is a transcription factor involved in de novo lipogenesis (DNL), which is, as its name suggests, responsive to glucose and other carbohydrates (33). ChREBP-β isoform (gene name *Mlxipl*, but referred to here as *ChrebpB*) mRNA expression was increased in L-Raptor-KO livers (Supplemental Figure 4A) and this expression corresponded with an induction in liver pyruvate kinase (*Pklr),* a transcriptional target of ChREBP-β, with no significant changes in xylulose-5-phosphate (34) (Supplemental Figure 4, B and C). Increased ChREBP-β activation is likely due to increased G6P levels (Figure 1E and Figure 2D), but would suggest that ChREBP-β acts as a transcriptional repressor of *Ppp1r3b*. Since ChREBP-β is classically considered a transcriptional activator of glycolytic and lipogenic genes, attention was directed to other feeding-regulated transcription factors.

*Srebp1c* was induced with feeding and its expression was significantly blunted in postprandial mTORC1-deficient livers, as reported previously (Figure 2A) (30, 35). Therefore, published cistromic and transcriptomic data were analyzed to determine if SREBP1c may act as transcription factor involved in *Ppp1r3b* expression. HA-nSREBP1c CHIP-Seq revealed SREBP1c binding near the transcription start site (TSS) of *Ppp1r3b* (36) (Supplemental Figure 5A). Furthermore, global run-on sequencing (GRO-Seq) data were examined to explore active sites of enhancers (27). Interestingly, an enhancer RNA (eRNA) in proximity of the *Ppp1r3b* gene colocalized with *Srebp1c* binding (Supplemental Figure 5A). These data suggest that *Srebp1c* may regulate the expression of *Ppp1r3b* postprandially. To test this, an AAV transcribing the nuclear form of SREBP1c (nSREBP1c), rendering the protein constitutively active, was coinjected with either AAV-GFP or CRE to generate control or overexpress nSREBP1c in L-Raptor-KO mice. Two weeks after AAV injection, nSREBP1c increased the lipogenic genes *Fasn* and *Acaca* but had no effect on *Gck* (Supplemental Figure 5B). Previous studies employing this AAV-nSREBP1c virus at the same dosage have validated its functional ability to restore DNL (36, 37). Reexpression of *nSrebp1c*, however, did not restore *Ppp1r3b* mRNA expression, nor did it restore postprandial hepatic glycogen content in the absence of liver mTORC1 (Supplemental Figure 5, C and D). Collectively, these data suggest that mTORC1 controlled the postprandial induction of *Ppp1r3b* and glycogen content in a mTORC1-dependent, SREBP1c-independent manner.

### mTORC1 activity is required for AKT-mediated inhibition of FOXO1 in the control of Ppp1r3b expression independent of Gck.

**Figure 5:**
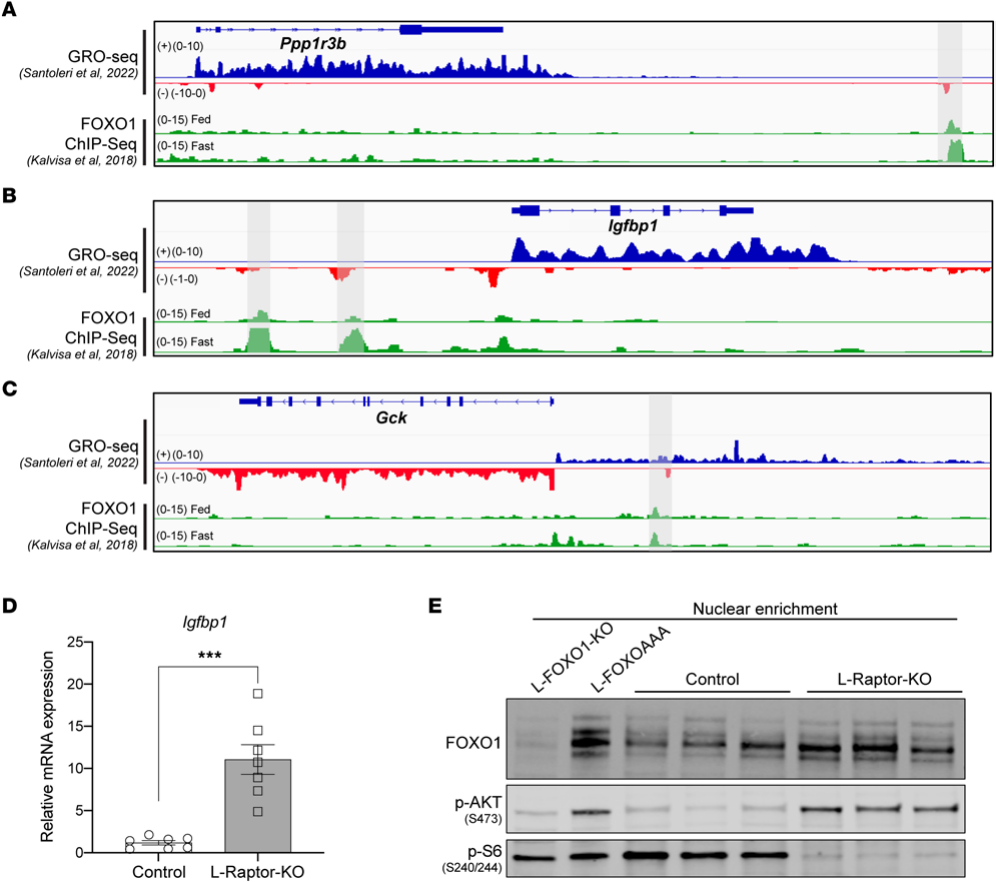
mTORC1 activity is required for AKT-mediated inhibition of FOXO1. (**A**–**C**) Genome browser track (mm9) GRO-Seq displaying *Ppp1r3b* and nearby eRNA corresponding with a FOXO1 ChIP-Seq track with previously identified FOXO1 binding highlighted in gray near genes (**A**) *Ppp1r3b,* (**B**) *Igfbp1,* and (**C**) *Gck*. (**D**) mRNA expression of *Igfbp1* in refed L-Raptor-KO livers. (**E**) Immunoblot of FOXO1 from refed liver lysates of control, L-Raptor-KO, L-FOXO1-KO, and L-FOXOAAA enriched for nuclear fraction. ***P < 0.001 versus control via Student’s *t* test. Data shown in ± SEM.

Since both SREBP1c and ChREBP-β were unlikely to be responsible for the transcriptional control of *Ppp1r3b*, our focus shifted to other feeding-regulated transcription factors implicated in glycemic control. Downstream of AKT, FOXO transcription factors are critical regulators of hepatic glucose production. During periods of fasting, FOXO proteins localize to the nucleus, where they promote transcription of gluconeogenic genes while recruiting corepressors to repress transcription of glucose utilization genes such as *Gck* (38). Upon feeding, AKT is activated and directly phosphorylates FOXO, excluding it from the nucleus, inhibiting its transcriptional regulatory functions. Analyzing our published GRO-Seq data set alongside a publicly available FOXO1 ChIP-Seq data set revealed FOXO binding occurs near an enhancer in proximity of *Ppp1r3b* (Figure 5A). FOXO1 binding is also identified at eRNAs localized near 2 canonical FOXO targets, insulin-like growth factor binding protein 1 (*Igfbp1)* and *Gck,* providing evidence that this ChIP-Seq reliably detected FOXO binding as a transcriptional activator and repressor, respectively (Figure 5, B and C).

**Figure 6:**
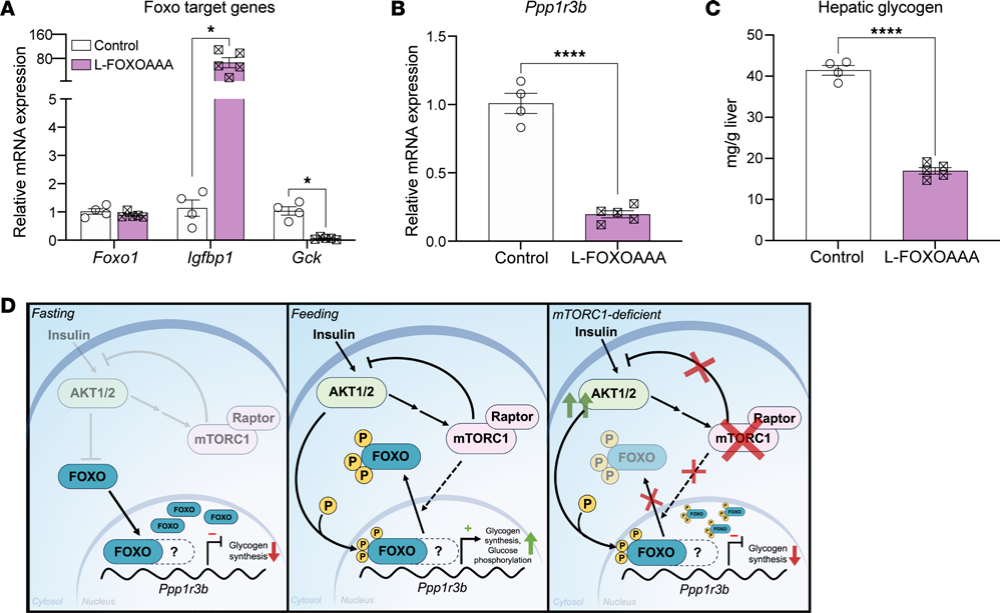
Activation of FOXO1 is required for Ppp1r3b repression. *Foxo1*^AAA^ mice aged 10–12 weeks were injected with AAV8-TBG-Cre (L-FOXOAAA) or AAV8-TBG-GFP (Control). Two weeks after injection, mice were fasted overnight, then refed chow for 4 hours before sacrifice. (**A**) Relative mRNA expression of FOXO target genes. (**B**) Relative mRNA expression of *Ppp1r3b*. (**C**) Hepatic glycogen levels in livers of mice reintroduced to food (refed). (**D**) Mechanistic schematic. Under fasting conditions, AKT and mTORC1 are inhibited, FOXO localizes to the nucleus where it recruits an unidentified corepressor (represented by the dashed line and ‘?’) to suppress transcription of *Ppp1r3b*, along with repression of *Gck*, to downregulate glycogen synthesis. Under feeding conditions, AKT facilitates phosphorylation of FOXO proteins and mTORC1 promotes the nuclear exclusion of AKT-phosphorylated FOXO (unknown mechanism represented by dashed arrow) to inhibit FOXO and promote transcription of *Ppp1r3b* and *Gck*. In the absence of mTORC1, AKT-phosphorylated FOXO proteins remain localized in the nucleus and continue to repress *Ppp1r3b* and *Gck*. *P < 0.05, ****P < 0.0001 versus indicated control via students *t* test. Data shown in ± SEM.

Although the mRNA levels of the FOXO1-regulated gene *Gck* are blunted in L-Raptor-KO (Figure 2B), we next tested whether *Igfbp1* mRNA expression was altered in the absence of mTORC1 activity as an additional readout of FOXO1 transcriptional activity. Notably, *Igfbp1* was significantly upregulated in L-Raptor-KO (Figure 5D), confirming increased FOXO activity. Nuclear enrichment of control and L-Raptor-KO livers revealed strong nuclear retention of FOXO1, despite increased AKT activity (Figure 5E). To determine if FOXO1 was sufficient to repress *Ppp1r3b*, we utilized a transgenic mouse model harboring a mutant FOXO1 with alanine substituting serine at the 3 AKT-mediated phosphorylation sites, leading to constitutive retention of FOXO1 in the nucleus (FOXO^AAA^) (39). To induce expression of the nuclear FOXOAAA mutant, specifically in hepatocytes, FOXO^AAA^ were injected with AAV8-TBG-CRE (L-FOXOAAA) or AAV8-TBG-GFP (Control) in 8-to-10-week-old mice and harvested livers 2 weeks after AAV injection. As predicted, constitutive FOXO1 activation in L-FOXOAAA mice yielded an induction in *Igfbp1* and a repression of *Gck* (Figure 6A). Notably, increased FOXO1 activity was sufficient to suppress *Ppp1r3b* and result in a significant blunting of postprandial hepatic glycogen content (Figure 6, B and C). Consistent with previous reports, these data indicate that mTORC1 activity is required for nuclear exclusion of AKT-mediated phosphorylated FOXO1, (40), and inhibition of FOXO is required for induction of *Ppp1r3b* and hepatic glycogen synthesis (Figure 6D).

Since GCK activity is also suppressed following activation of FOXO1, we next determined if GCK was required for feeding-induced *Ppp1r3b* expression. To do so, we used a mouse model lacking GCK in hepatocytes. *Gck*^loxP/loxP^ mice were injected with AAV8-TBG-CRE (L-GCK-KO) or AAV8-TBG-GFP (Control) where detection of GCK protein and mRNA was lost (Figure 2C and Supplemental Figure 6A). In the absence of hepatic GCK, *Ppp1r3b* mRNA levels remained unchanged, revealing that GCK signaling was not required to induce *Ppp1r3b* (Supplemental Figure 6B). Collectively, these data indicate a requirement for mTORC1 in FOXO1 nuclear exclusion and inhibition and suggest that both AKT and mTORC1 activity were required but not sufficient to control hepatic FOXO1 activity and glycogen accumulation.

## Discussion

The data presented in this manuscript demonstrate a requirement for mTORC1 in postprandial glycogen synthesis. Here, we demonstrate that mTORC1 controls the feeding induction of *Ppp1r3b* to regulate GS activity and glycogenesis through inhibition of FOXO1. Furthermore, we identify differential changes in metabolite pools as the liver transitions from a nutrient-deprived to a nutrient-abundant feeding state. Taken together, these findings highlight an essential role for mTORC1 in hepatic glucose metabolism and highlight the importance of *Ppp1r3b* in glycogen homeostasis and transcriptional mechanisms governing its molecular regulation.

Deletion of the liver-specific isoform of glycogen synthase (*Gys2)* in mice results in an almost complete depletion of liver glycogen content (41). Additionally, human loss-of-function mutations that cause a deficiency in glycogen synthase, specifically GSD Type 0 (GSD0), have depleted glycogen stores in the liver (42). Taken together, GS activity is an essential regulator of hepatic glycogen content. A potent regulator of GS activity is PP1, including the hepatic G_L_ subunit, PPP1R3B. As previously noted, genetic variations near the *PPP1R3B* locus are associated with fasting insulin and fasting glucose, as characterized in the MAGIC study (20). Although we detect modest changes in phospho-GS at Serine-641 that correspond with robust functional changes in GS activity, additional phosphorylation sites on GS such as Ser8 may also be regulated by this mTORC1-PPP1R3B axis (43). Therefore, it is critical to understand the postprandial mechanisms regulating *Ppp1r3b* expression and GS activity. With this study, we believe that we have added mechanistic insight into how nutrient intake regulates *Ppp1r3b* and GS activity, contributing to postprandial glycogen synthesis.

Despite many studies using rapamycin as a pharmacological approach to inhibit mTORC1, many fail to report how rapamycin impacts glycogen levels in the liver. However, existing evidence suggests that rapamycin treatment blunts GS activity and glycogen synthesis. In a canine model of GSDIIIa, rapamycin treatment downregulated total hepatic glycogen content (24). In skeletal muscle models, rapamycin treatment led to increased GS phosphorylation, consistent with decreased GS activity, and further blunting of insulin-stimulated glycogen synthesis (44, 45). Long-term rapamycin use is linked to impaired glucose tolerance and insulin sensitivity in rodent models (46, 47). Chronic administration of rapamycin also inhibits mTORC2-AKT making it difficult to isolate the specific effects of hepatic mTORC1 in the control of glycogen content (28), highlighting the importance of this study, in that it specifically isolates the role of hepatic mTORC1 signaling on glycogen synthesis and glucose homeostasis.

Our data demonstrate the requirement of mTORC1 signaling for glycogen synthesis via acute liver-specific deletion of the Raptor protein. Other data involving constitutive activation of mTORC1, via deletion of the negative regulator of mTORC1, TSC, support these findings. In *Tsc2*-deficient MEFs, intracellular glycogen accumulated to higher levels above control cells, which was reversed with rapamycin treatment or Raptor knockdown (26). Human loss-of-function mutations in tuberous sclerosis complex (TSC), for example, cardiac rhabdomyomas, present with excess glycogen deposition, correlating with increased mTORC1 activity (48, 49). Similarly, in mice lacking TSC1 in ventricular myocytes, myocytes had unrestrained mTORC1 activity and accumulated glycogen (50). Collectively, data from TSC studies support mTORC1 activation, promoting glycogenesis in cells other than hepatocytes. However, it is challenging to interpret data from TSC-deficient models, as constitutive activation negatively impacts AKT activity, and AKT is required for postprandial hepatic glycogen storage (22, 37, 51, 52). This is consistent with studies that show loss of TSC in hepatocytes decreases hepatic glycogen content, which is likely due to the downregulation of AKT signaling that occurs in little as 2 weeks following TSC deletion in adult liver (53, 54). Collectively, these data indicate that mTORC1 is required, but not sufficient, to control hepatic glycogenesis.

Mechanistically, our data demonstrate the requirement for mTORC1 activity in the nuclear exclusion and inhibition of FOXO1 in the induction of *Ppp1r3b* and glycogen synthesis. Previous studies demonstrate nuclear accumulation of AKT-phosphorylated FOXO in the absence of mTORC1 activation (40). Further, our data correspond with prior observations of hepatic glycogen accumulation following FOXO inhibition (55). These data illustrate the requirement for mTORC1 in the canonical AKT-dependent inhibition of FOXO proteins (Figure 6G) and that phosphorylation of FOXO1 via AKT is not sufficient to drive nuclear export in the absence of mTORC1. Understanding the complex molecular interplay between AKT and mTORC1 in the regulation of FOXO1 is critical to our understanding of hepatic metabolism and will be the focus of future studies.

In summary, we provide evidence for a feeding-dependent mechanism of GS regulation and glycogenesis through *Ppp1r3b* via the nutrient-sensing kinase mTORC1. These data provide additional mechanistic insight into the molecular control of postprandial glucose metabolism and provide important physiological context to the molecular regulation of a T2D gene, *Ppp1r3b*. Elucidating the postprandial mechanisms of glucose disposal is vital for our understanding of liver physiology in health and diseases such as insulin resistance and T2D.

## Methods

### Sex as a biological variable.

This study exclusively examined male mice, however we expect similar results in female mice, due to the well-established role of mTORC1 in hepatic metabolism.

### Animal experiments.

*Rptor*^loxp/loxp^, *Gck*^loxp/loxp^, *Foxo1*^loxp/loxp^, and *Foxo1*^AAA^ (otherwise known as R26StopFlFoxo1^AAA^) mice were backcrossed to the C57BL/6J background (39, 52, 56, 57), housed, and bred under specific pathogen–free conditions in facilities at the University of Pennsylvania. For acute excision of liver-specific genes, mice were injected with AAV (Vector Core, University of Pennsylvania) containing a liver-specific thyroxine binding globulin (TBG) promoter serotype 8 (AAV8-TBG) containing either GFP (AAV-GFP) or Cre (AAV-Cre) at a dosage of 1.0 × 10^11^ genome copies. AAV8-TBG-nSREBP1c virus was a kind gift from Dr. Mitchell Lazar (University of Pennsylvania). All mice were fed chow diet (LabDiet, no. 5010) unless specified otherwise. Control animals consisted of pools of the appropriate floxed mice for each experiment, *Rptor*^loxp/loxp^, which were injected with AAV-GFP. Mice that were coinjected with AAV-Ppp1r3b or AAV-nSREBP1c and AAV-cre received 3.0 × 10^11^ and 1.0 × 10^11^ genome copies, respectively, for a total of 4.0 × 10^11^ genome copies in a single injection. Consistently, control mice were injected with 4.0 × 10^11^ genome copies of AAV-GFP, and L-Raptor-KO mice were injected with 1.0 × 10^11^ and 3.0 × 10^11^ of AAV-Cre and AAV-GFP, respectively, for a total injection of 4.0 × 10^11^ genome copies. All experiments were performed in male mice.

### Tissue metabolite extraction.

Mice were euthanized by cervical dislocation. Tissues were quickly dissected and snap frozen in liquid nitrogen with a precooled clamp. Snap-frozen tissues were transferred to 2 mL round-bottom Eppendorf safe-lock tubes on dry ice. Samples were then ground into powder with a cryomill machine (21) for 30 seconds at 25 Hz and maintained at a cold temperature using liquid nitrogen. For every 20 mg tissue, 800 μL −20°C 40:40:20 (v/v/v) acetonitrile:methanol:water (Thermo Fisher Scientific) solution was added to the tube, vortexed for 10 seconds, and then centrifuged at 21,000*g* for 20 minutes at 4°C. The supernatants were then transferred to plastic vials for LC-MS analysis. A procedure blank was generated identically without tissue and was used later to remove the background ions.

### Plasma metabolite extraction.

Plasma (2.5 μL) was added to 60 μL −20 °C 25:25:10 (v/v/v) acetonitrile:methanol:water solution, vortexed for 10 seconds, and put on ice for at least 5 minutes. The resulting extract was centrifuged at 21,000*g* for 20 minutes at 4°C and supernatant was transferred to tubes for LC-MS analysis. A procedure blank was generated identically without plasma, which was used later to remove the background ions.

### Metabolite measurement by LC-MS.

Metabolites were analyzed using a Vanquish Horizon UHPLC System (Thermo Fisher Scientific) coupled to an Orbitrap Exploris 480 mass spectrometer (Thermo Fisher Scientific). Waters XBridge BEH Amide XP Column [particle size, 2.5 μm; 150 mm (length) × 2.1 mm (i.d.)] was used for hydrophilic interaction chromatography (HILIC) separation. Column temperature was kept at 25°C. Mobile phases A = 20 mM ammonium acetate and 22.5 mM ammonium hydroxide in 95:5 (v/v) water:acetonitrile (pH 9.45) and B = 100% acetonitrile were used for both ESI positive and negative modes. The linear gradient eluted from 90% B (0.0–2.0 minutes), 90% B to 75% B (2.0–3.0 minutes), 75% B (3.0–7.0 minutes), 75% B to 70% B (7.0 –8.0 minutes), 70% B (8.0–9.0 minutes), 70% B to 50% B (9.0–10.0 minutes), 50% B (10.0–12.0 minutes), 50% B to 25% B (12.0–13.0 minutes), 25% B (13.0 –14.0 minutes), 25% B to 0.5% B (14.0–16.0 minutes), 0.5% B (16.0–20.5 minutes), then stayed at 90% B for 4.5 minutes. The flow rate was 0.15 mL/min. The sample injection volume was 5 μL. ESI source parameters were set as follows: spray voltage, 3,200 V or −2,800 V, in positive or negative modes, respectively; sheath gas, 35 arb; aux gas, 10 arb; sweep gas, 0.5 arb; ion transfer tube temperature, 300 °C; vaporizer temperature, 35°C. LC–MS data acquisition was operated under a full-scan polarity switching mode for all samples. The full scan was set as orbitrap resolution, 120,000 at m/z 200; AGC target, 1× 10^7^; maximum injection time, 200 ms; scan range, 60–1,000 m/z.

### Data analysis.

LC-MS raw data files (.raw) were converted to mzXML format using ProteoWizard (version 3.0.20315). El-MAVEN (version 0.12.0) was used to generate a peak table containing m/z, retention time, and intensity for the peaks. Parameters for peak picking were the defaults except for the following: mass domain resolution, 5 ppm; time domain resolution, 10 scans; minimum intensity, 10,000; and minimum peak width, 5 scans. The resulting peak table was exported as a.csv file. Peak annotation of untargeted metabolomics data was performed using NetID with default parameters. For tracer experiments, isotope labeling was corrected for ^13^C natural abundances using AccuCor package.

### Immunoblots.

Protein lysates were prepared from frozen livers in a modified RIPA buffer with Phosphatase Inhibitor Cocktails 2 and 3 (Sigma-Aldrich) and cOmplete Protease Inhibitor Cocktail (58), as described previously (29). The following antibodies were used for immunoblotting, all from Cell Signaling Technology: p-AKT (CST no. 4060), AKT2 (CST no. 2964), p-S6 (CST no. 2215), S6 (CST no. 2217), HSP90 (CST no. 4874), GCK (Gift from Magnuson Lab), p-GS (CST no. 3891), p-GSK3β (CST no. 9336), Raptor (CST no. 2280), and FOXO1 (CST no. 9454).

### mRNA isolation and real-time PCR.

Total RNA was isolated from frozen livers using the RNeasy Plus kit (Qiagen). Complementary DNA was synthesized using Moloney murine leukemia virus (MulV) reverse transcriptase, and the relative expression of the genes of interest was quantified by real-time PCR using the SYBR Green dye-based assay.

### Histology.

Livers were fixed in 10% buffered formalin overnight, dehydrated in ethanol, paraffin-embedded, and sectioned. Sections were stained with H&E or PAS staining.

### Liver glycogen determination.

Glycogen was extracted from 100 mg of liver in 6% perchloric acid by digesting the samples in KOH followed by digestion with amylo-glucosidase (Sigma-Aldrich). Resulting free glycosyl units were assayed spectrophotometrically using a hexokinase-based glucose assay kit (Sigma-Aldrich) and compared with the glucose levels in the samples before enzymatic digestion.

### GS activity assay.

Liver tissues were weighed and homogenized in 1:20 (wet) mass/mL ice cold lysis buffer (270 mM sucrose, 50 mM Tris-HCl (pH 7.5), 1 mM EDTA, 1 mM EGTA, 1% (v/v) Triton X-100, 20 mM glycerol-2-phosphate, 50 mM NaF, 5 mM Na4P2O7, 1 mM DTT, 0.1 mM PMSF, 1 mM benzamidine, 1 mg/mL microcystin-LR, 2 mg/mL leupeptin, and 2 mg/mL pepstatin A [Sigma-Aldrich]), followed by centrifugation at 3000*g* for 5 minutes at 4°C. GS activity in the liver lysates was determined as described previously (8). Briefly, clarified lysates were diluted to a concentration of 2.5 mg/mL with ice cold lysis buffer in a total volume of 100 μL. 20 μL of the protein solution was added to 80 μL of the assay buffer (25 mM Tris-HCl (pH 7.8), 50 mM NaF, 5 mM EDTA, 10 mg/ml glycogen), 5.5 mM UDP-glucose, 12.5 m-m Na_2_SO_4_, 0.125% (v/v) β-mercaptoethanol and 0.05 mCi/mmol or 0.15 mCi/mmol D-[^14^C]-UDP-glucose (American Radiolabeled Chemicals,Inc., ARC 0154) with or without 12.5 mM G6P. Note: 0.05 mCi/mmol D-[^14^C]-UDP-glucose was used for the samples incubated in the presence of G6P and 0.15 mCi/mmol D-[^14^C]-UDP-glucose was used for the samples incubated in the absence of G6P (due to very low basal GS activity in the liver). The reaction mixtures were incubated for 30 minutes at 30 °C with mild agitation at 300 rpm. The reactions were stopped by spotting 90 μL of the reaction mix onto 2.5 cm × 2.5 cm squares of filter paper (Whatman 3MM) which were immediately immersed in ice cold 66% ethanol and left to incubate with mild agitation for 20 minutes. The filter papers were washed thrice more in 66% ethanol for 20 minutes each and rinsed in acetone. The dried filters were subjected to scintillation counting.

### Statistics.

Statistical analysis was performed using 1-way ANOVAs when more than 2 groups were compared, 2-way ANOVAs when 2 conditions were analyzed, and unpaired 2-tailed Student’s *t* test when 2 groups were being assayed. All data were presented as mean ± SEM. *P value < 0.05, **P value < 0.01, ***P value < 0.001, ****P value < 0.0001 versus indicated genotype.

### Study approval.

Animal use followed all standard and guidelines and were approved by the Institutional Animal Care and Use Committee (IACUC) at the University of Pennsylvania.

### Data availability.

Data available in the Supporting Data Values file.

## Author contributions

KU conceived the hypothesis, designed and performed experiments, analyzed data, and prepared the manuscript. WDL provided technical assistance, contributed to experimental design, and analyzed data. WQ and DS designed and performed experiments and analyzed data. DB performed experiments and analyzed data. MS, TC, and AGW provided technical assistance. KTC and JDR contributed conceptually to experimental design and data analysis. KS contributed to experimental design and analyzed data. DJR provided virus and contributed to experimental design. PMT conceived the hypothesis, designed and performed experiments, analyzed data, prepared the manuscript, and directed the project.

## Supplementary Material

Supplemental data

Unedited blot and gel images

Supporting data values

## Version 1 01/30/2024

In-Press Preview

## Version 2 04/01/2024

Electronic publication
