# Supplementary material for: mTORC1 controls murine postprandial hepatic glycogen synthesis via Ppp1r3b
Source: J Clin Invest. 2024 Jan 30;134(7):e173782. doi: 10.1172/JCI173782 (PMC10977990; doi:10.1172/JCI173782)
Supplement: Supplemental data [file jci-134-173782-s025.pdf]

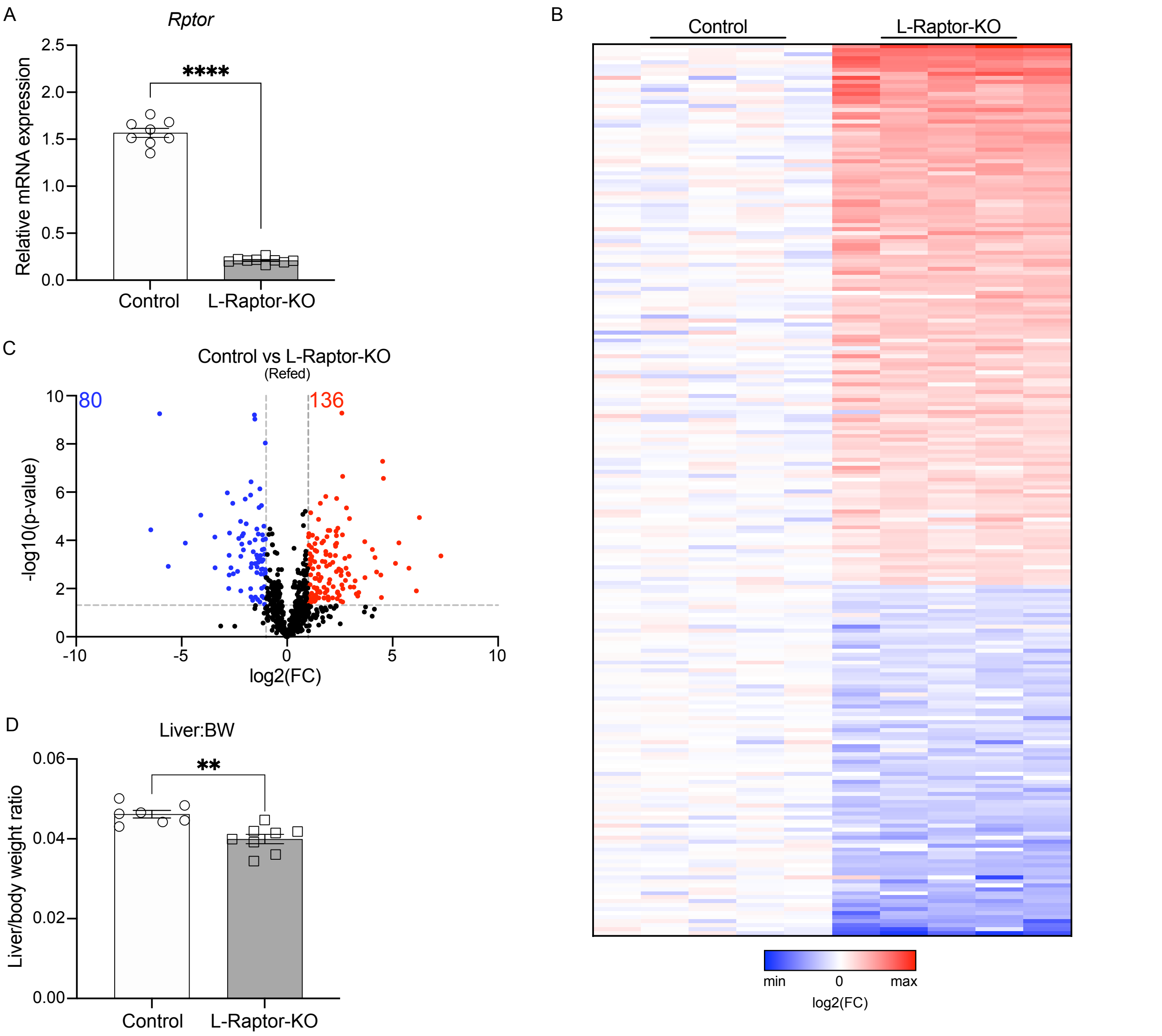

Supplemental Figure 1: Postprandial metabolomics of mTORC1-deficient livers
10-12 week old *Rptor*$^{loxP/loxP}$ mice were injected with AAV8-TBG-Cre (L-Raptor-KO) or AAV8-TBG-GFP (Control). Two weeks after injection, mice were fasted overnight, then refed chow for 4 hours before sacrifice. (A) Relative mRNA expression of *Rptor* gene. (B) Heat map of differential metabolite abundance shown as log2(fold change) compared to control livers, where log2(FC)>2 or log2(FC)<-2. (C) Volcano plot showing -log10(p-value vs. control refed) on y-axis and log2(fold change vs control refed) on x-axis. Blue dots represent log2(FC)<-2, p<0.01. Red dots represent log2(FC)>2, and p<0.01. (D) Liver weight (grams) to body weight (grams) ratio. **p<0.01, ****p<0.0001 vs control via students t-test.Data shown as mean +/- SEM.v Red indicates higher metabolite abundance.

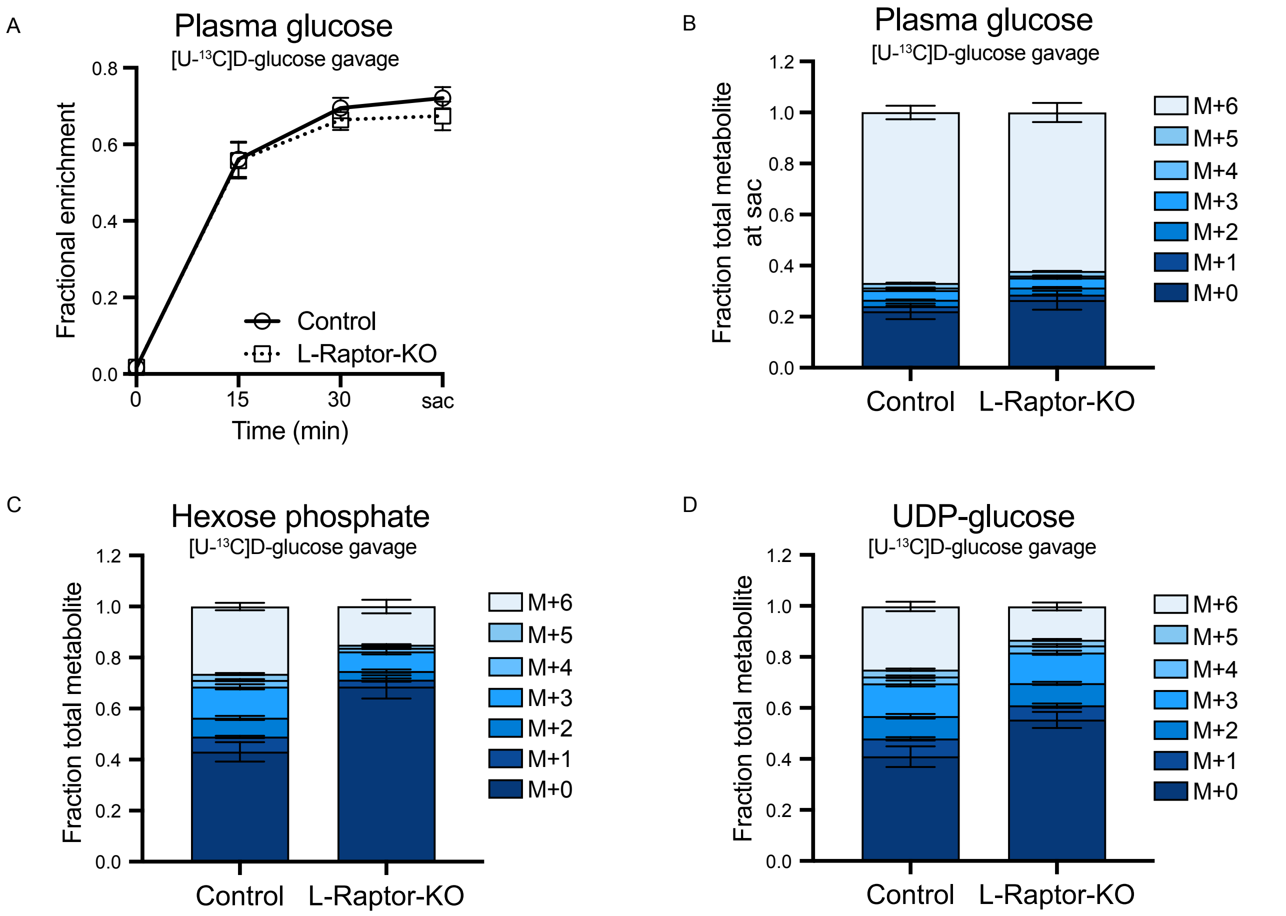

Supplemental Figure 2: mTORC1 is required for hepatic glycogen synthesis
(A,B) 10-12 week old *Rptor*$^{loxP/loxP}$ mice were injected with AAV8-TBG-GFP (Control), AAV8-TBG-Cre in combination with AAV8-TBG-GFP (L-Raptor-KO). 2 weeks after AAV injection, mice were fasted overnight and subjected to oral gavage with 2g/kg U-13C-D-glucose. Mice were sacrificed and livers were harvested 30 minutes after oral gavage. (A,B) Labeled plasma glucose measured at T0, T15, T30, and at harvest (sac). Fractional enrichment over time and at harvest indicate no difference in glucose labeling between Control and L-Raptor-KO mice. (C,D) Fractional labeling of hexose phosphate and UDP-glucose, respectively. Data shown as mean +/- SEM.

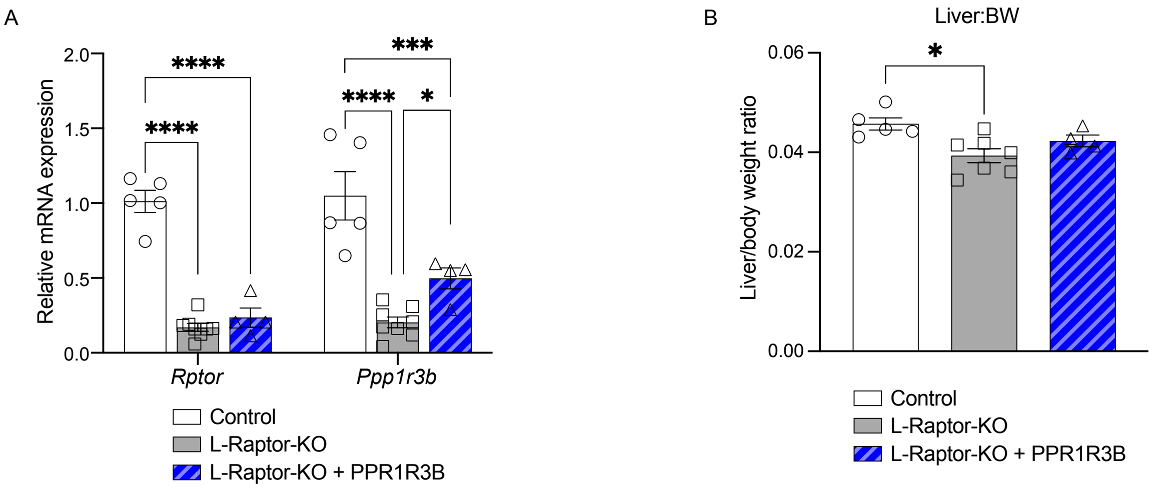

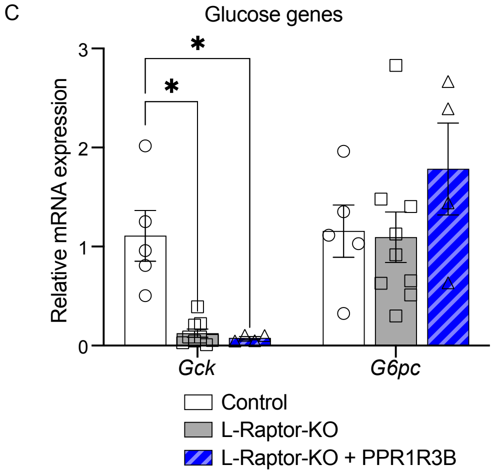

Supplemental Figure 3: Restoration of *Ppp1r3b* in L-Raptor-KO livers promotes GS activity and glycogen storage

(A,B) 10-12 week old *Rptor*$^{loxP/loxP}$ mice were injected with AAV8-TBG-GFP (Control), AAV8-TBG-Cre in combination with AAV8-TBG-GFP (L-Raptor-KO), or AAV8-TBG-Cre in combination with AAV8-TBG-Ppp1r3b (L-Raptor-KO + PPP1R3B), two weeks prior to an overnight fast and 4 hour refed (see Figure 4A for experimental design). (A) Relative gene expression of *Rptor* and *Ppp1r3b* gene. (B) Liver weight (grams) to body weight (grams) ratio. (C) Relative mRNA expression for *Gck* (glucokinase) and *G6pc* (glucose-6-phosphatase). *p<0.05, ***p<0.001, ****p<0.0001 via one-way ANOVA. Data shown as mean +/- SEM.

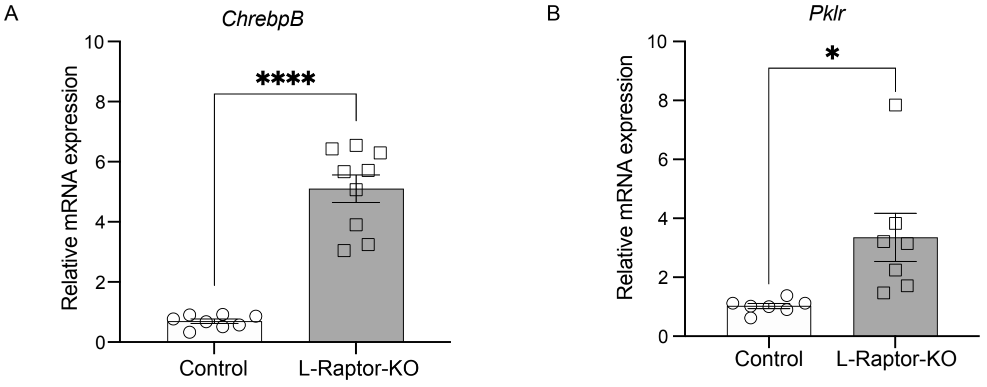

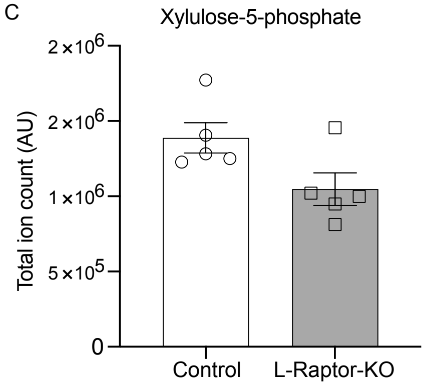

Supplemental Figure 4: ChrebpB is activated in mTORC1-deficient livers

(A,B) 10-12 week old *Rptor*$^{loxP/loxP}$ mice were injected with AAV8-TBG-GFP (Control), AAV8-TBG-Cre in combination with AAV8-TBG-GFP (L-Raptor-KO), or AAV8-TBG-Cre in combination with AAV8-TBG-Ppp1r3b (L-Raptor-KO + PPP1R3B), two weeks prior to an overnight fast and 4 hour refed (see Figure 4A for experimental design). (A,B) *ChrebpB* and *Pklr* (liver pyruvate kinase) mRNA expression. (C) Ion count of xylulose-5-phosphate in refed livers. *p<0.05, ****p<0.0001 students t-test. Data shown as mean +/- SEM.

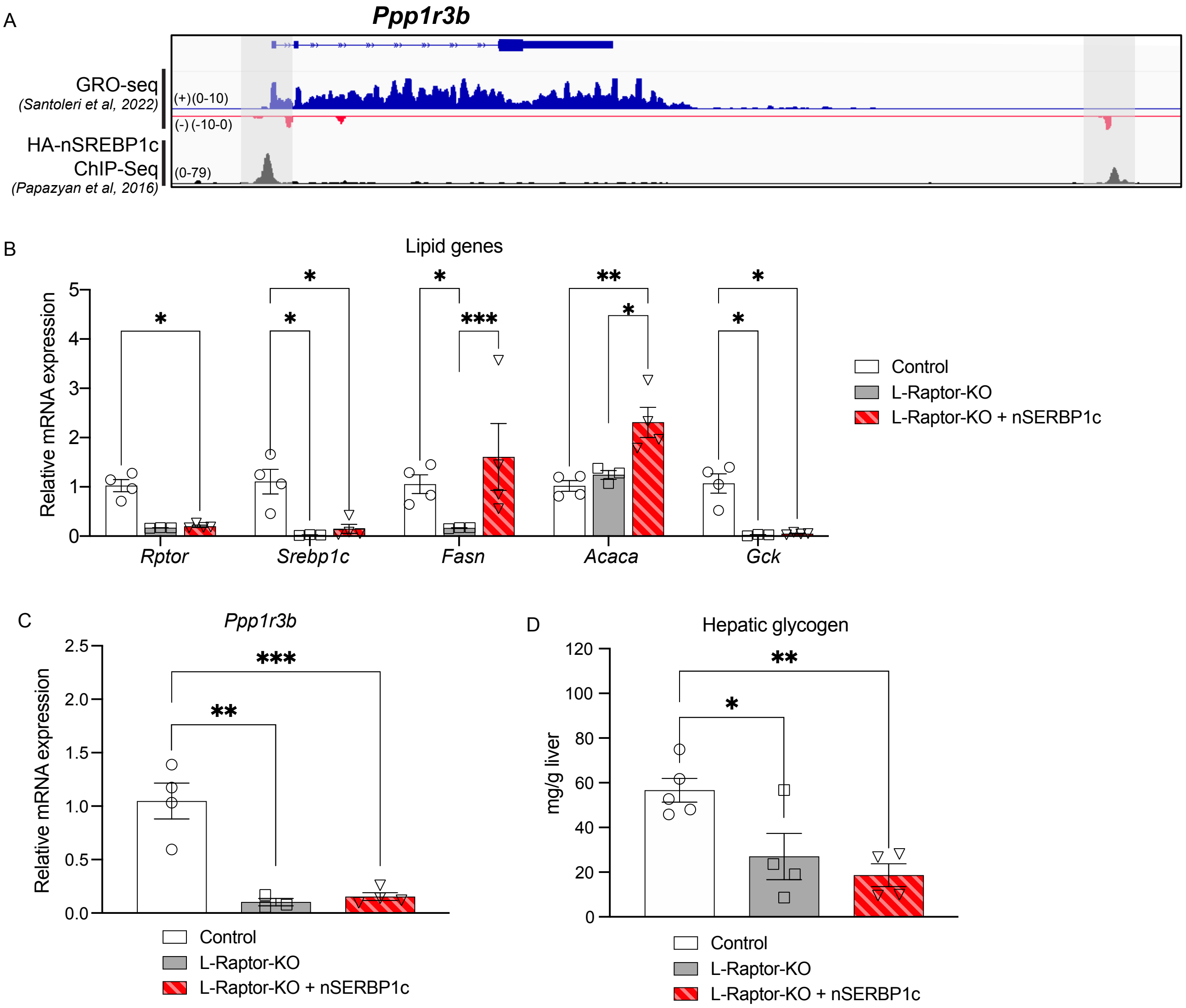

Supplemental Figure 5: Exogenous SREBP1c expression fails to restore hepatic glycogen in L-Raptor-KO mice
(A) Genome browser track (mm9) GRO-seq displaying *Ppp1r3b* and nearby eRNA corresponding with an HA-Srebp1c ChIP-seq track. Potential Srebp1c binding sites are highlighted in grey. (B-D) 8-10 week old *Rptor*$^{loxP/loxP}$ mice were injected with AAV8-TBG-GFP (Control), AAV8-TBG-Cre in combination with AAV8-TBG-GFP (L-Raptor-KO) or AAV8-TBG-Cre in combination with AAV8-TBG-nSREBP1c (L-Raptor-KO + nSREBP1c) 2 weeks prior to an overnight fast and 4 hour refed. (B) Relative gene expression of SREBP1 target genes (lipid synthesis related genes). (C) Relative mRNA expression of *Ppp1r3b*. (D) Hepatic glycogen in fed livers. *p<0.05, **p<0.01, ***p<0.001 vs indicated genotype via one-way ANOVA. Data shown in +/- SEM.

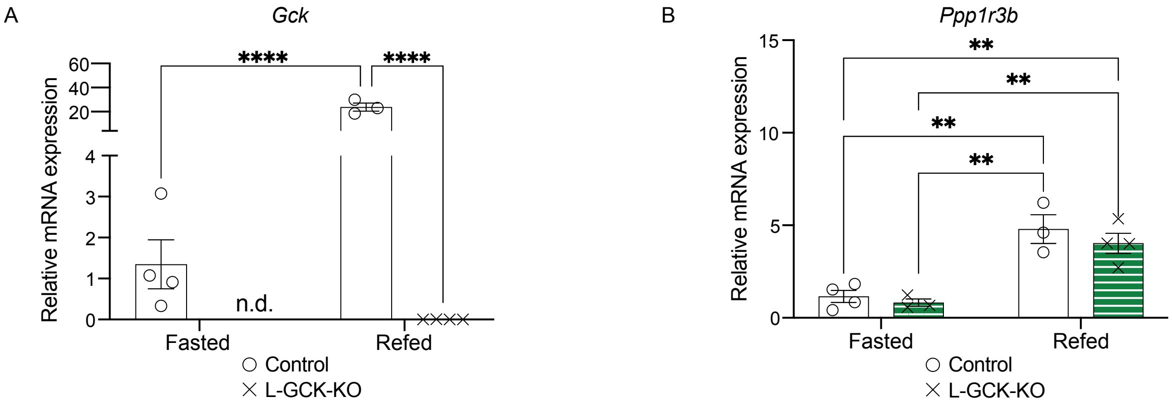

Supplemental Figure 6: GCK activity is not required for Ppp1r3b induction and not sufficient for postprandial glycogen storage
8-10 week old *Gck*$^{loxP/loxP}$ mice were injected with AAV8-TBG-GFP (Control), AAV8-TBG-Cre (L-GCK-KO). Mice were either fasted overnight or refed for 4 hours. Relative mRNA expression of *Gck* and *Ppp1r3b*. **p<0.01, ****p<0.0001 vs. indicated genotype via 2-way ANOVA. Data shown in +/- SEM.
