# Supplementary material for: mTORC1 controls murine postprandial hepatic glycogen synthesis via Ppp1r3b
Source: J Clin Invest. 2024 Jan 30;134(7):e173782. doi: 10.1172/JCI173782 (PMC10977990; doi:10.1172/JCI173782)
Supplement: Unedited blot and gel images [file jci-134-173782-s026.pptx]

## Slide 1
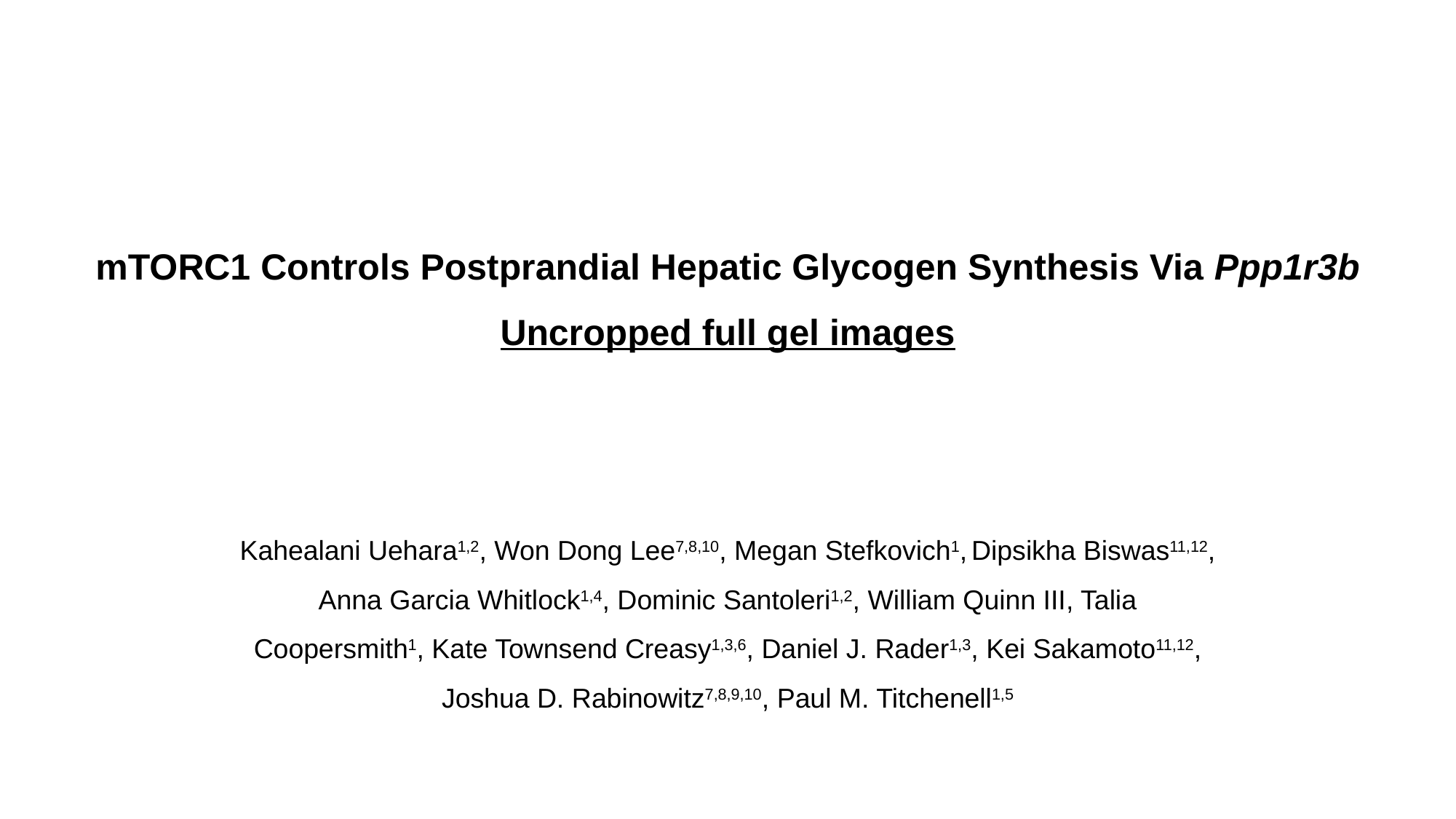

mTORC1 Controls Postprandial Hepatic Glycogen Synthesis Via Ppp1r3b
Uncropped full gel images
Kahealani Uehara1,2, Won Dong Lee7,8,10, Megan Stefkovich1, Dipsikha Biswas11,12, Anna Garcia Whitlock1,4, Dominic Santoleri1,2, William Quinn III, Talia Coopersmith1, Kate Townsend Creasy1,3,6, Daniel J. Rader1,3, Kei Sakamoto11,12, Joshua D. Rabinowitz7,8,9,10, Paul M. Titchenell1,5

## Slide 2
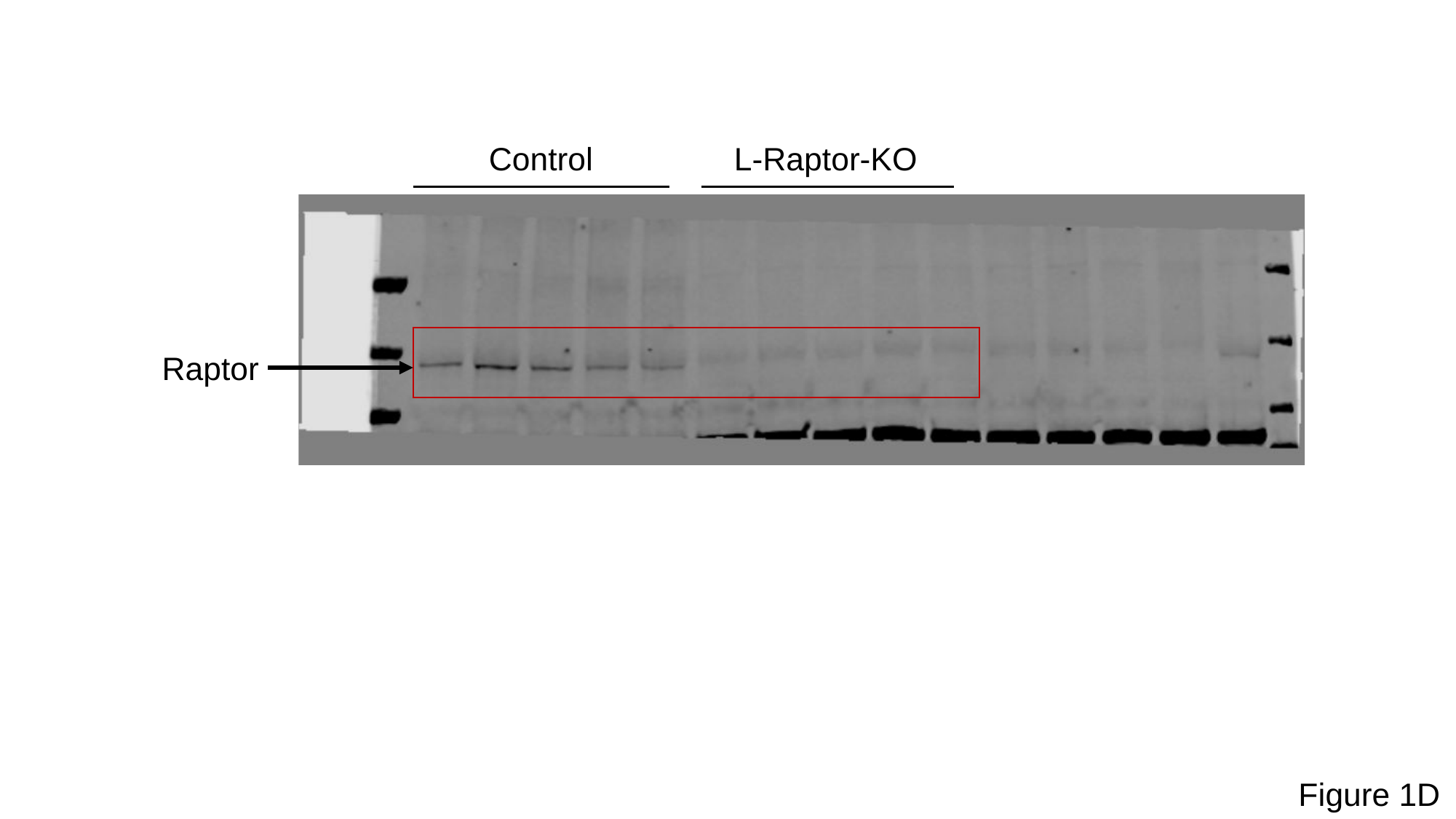

Control
L-Raptor-KO
Raptor
Figure 1D

## Slide 3
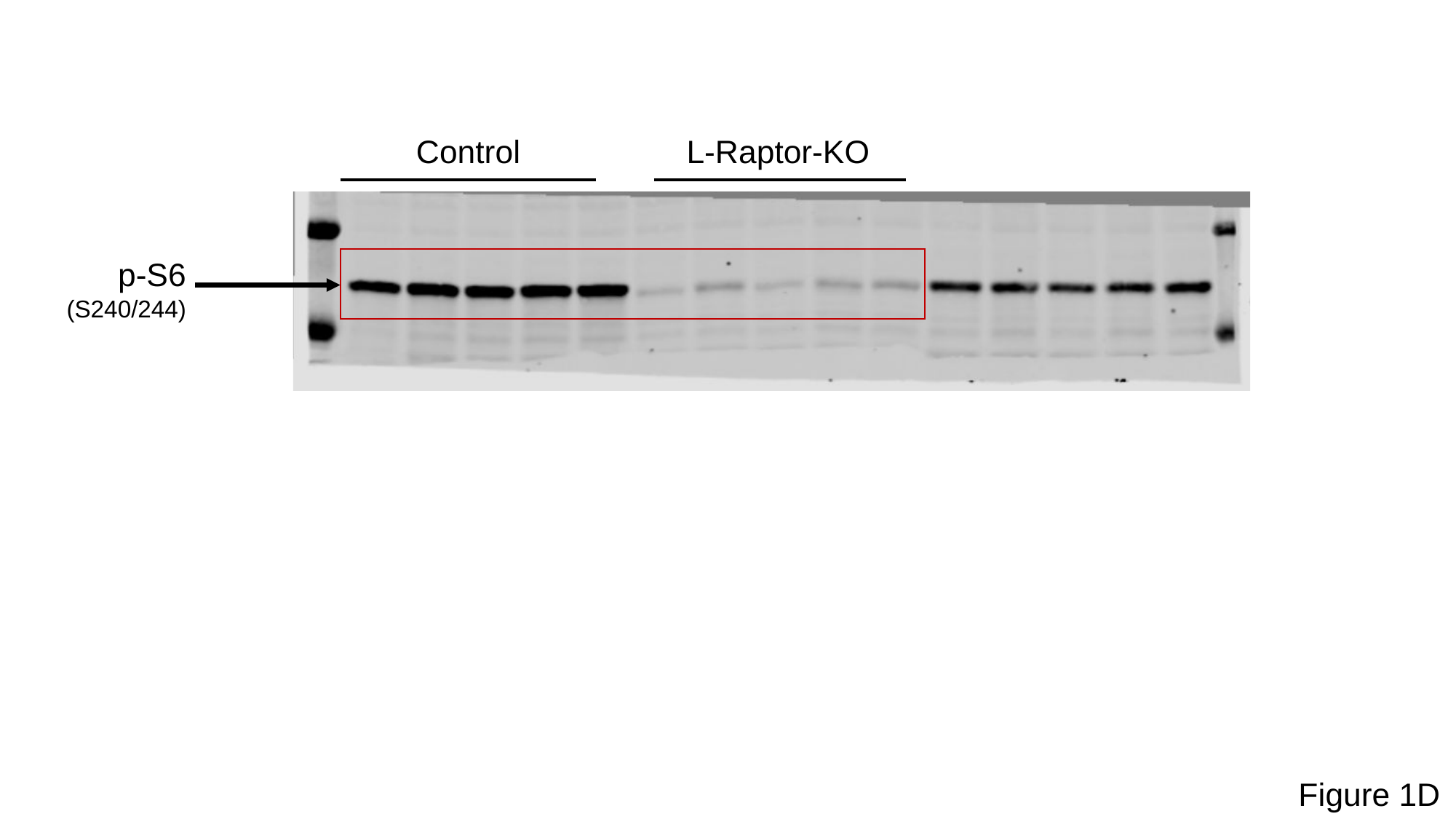

Control
L-Raptor-KO
p-S6
(S240/244)
Figure 1D

## Slide 4
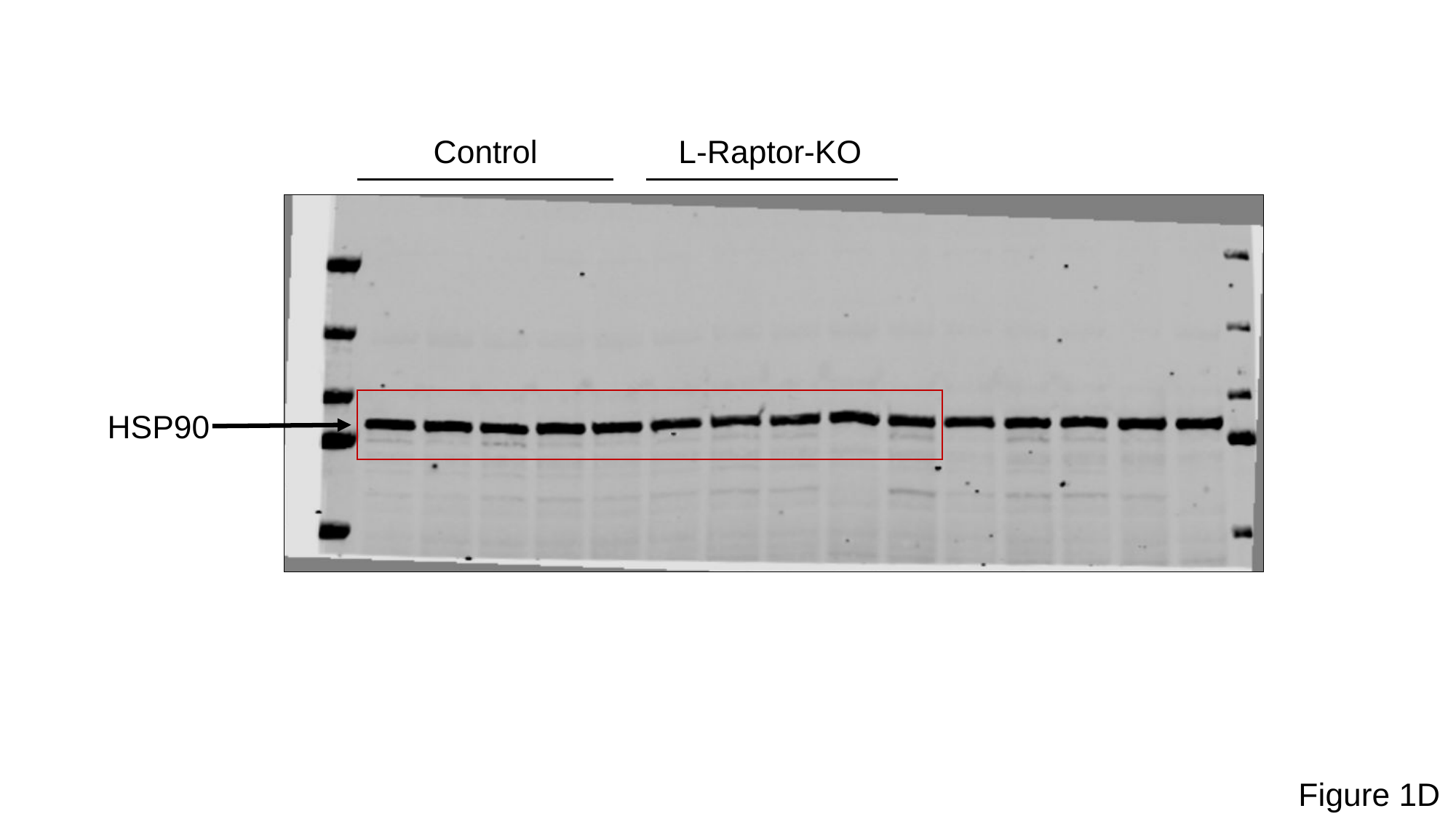

Control
L-Raptor-KO
HSP90
Figure 1D

## Slide 5
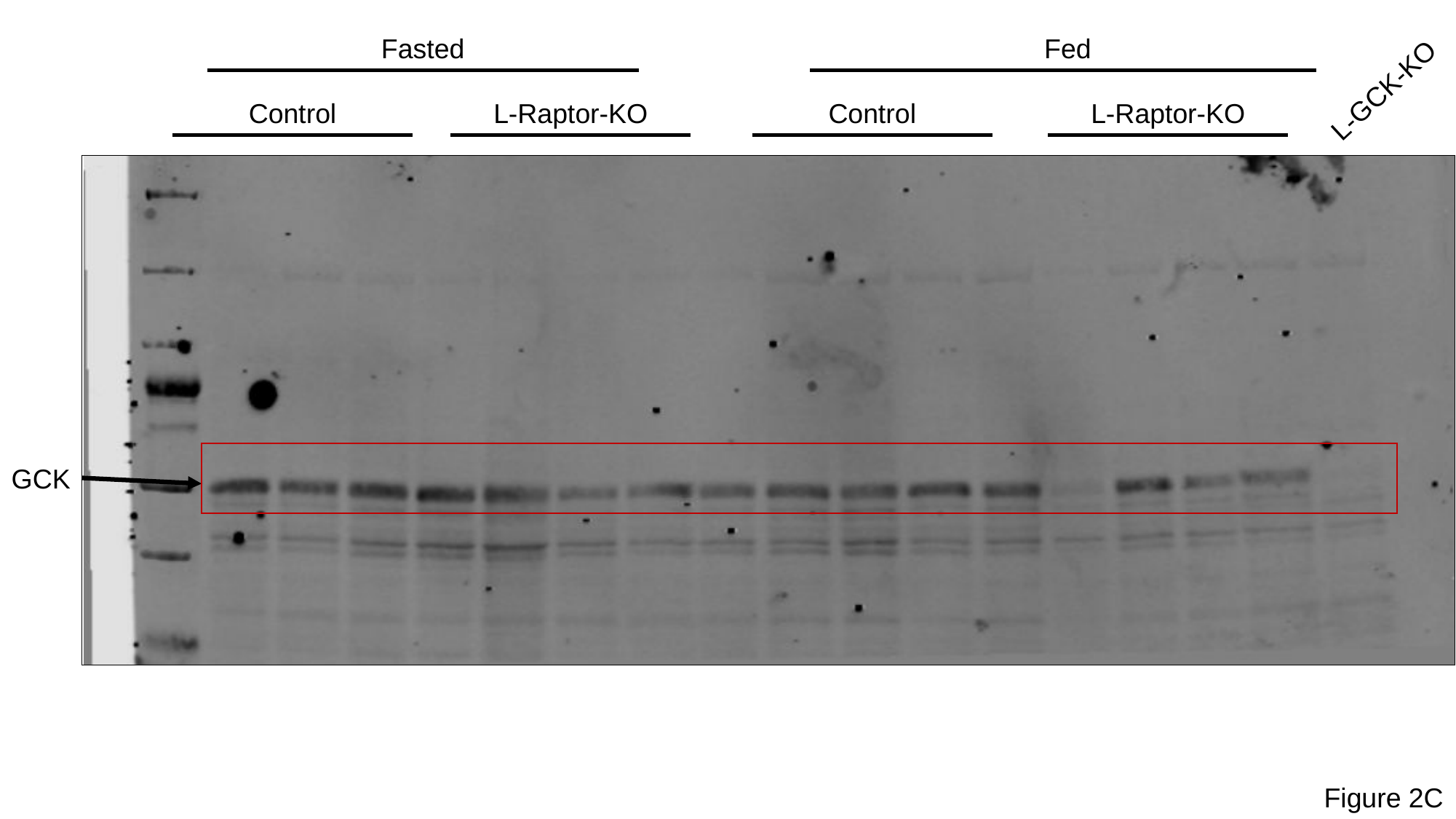

Fasted
Fed
L-GCK-KO
Control
L-Raptor-KO
Control
L-Raptor-KO
GCK
Figure 2C

## Slide 6
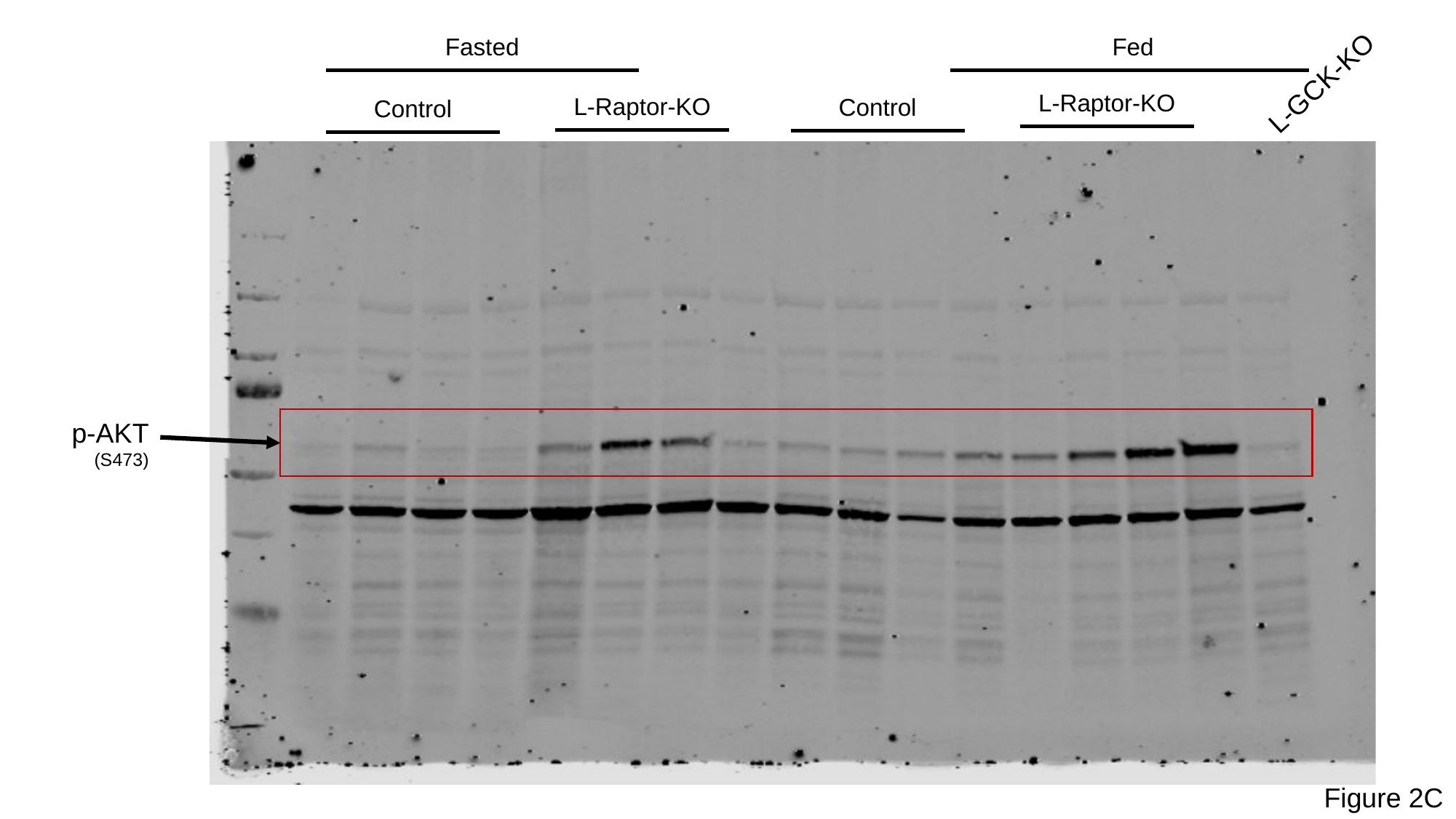

Fasted
Fed
L-GCK-KO
L-Raptor-KO
L-Raptor-KO
Control
Control
p-AKT
(S473)
Figure 2C

## Slide 7
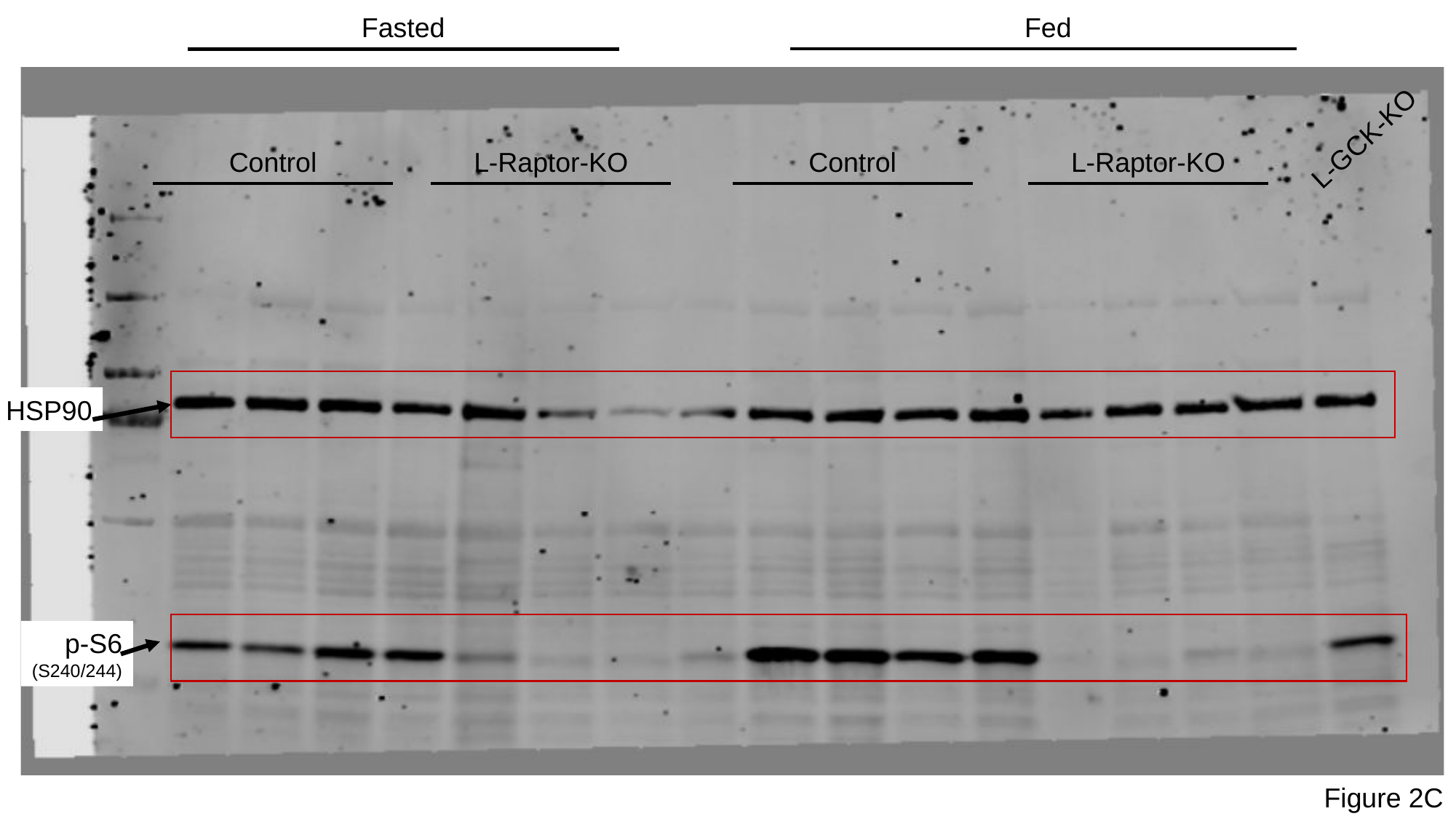

Fasted
Fed
L-GCK-KO
Control
L-Raptor-KO
Control
L-Raptor-KO
HSP90
p-S6
(S240/244)
Figure 2C

## Slide 8
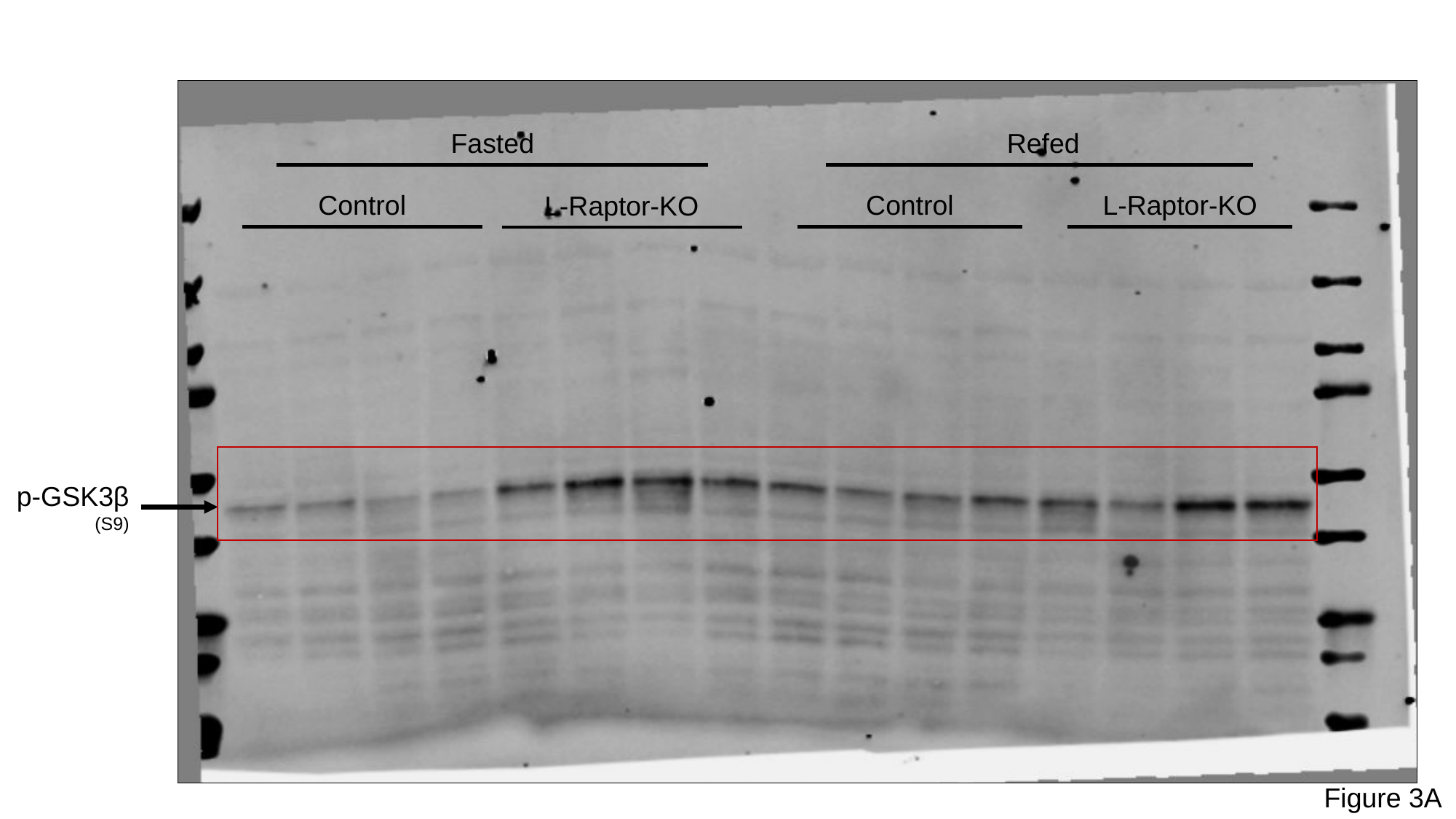

Fasted
Refed
Control
Control
L-Raptor-KO
L-Raptor-KO
p-GSK3β
(S9)
Figure 3A

## Slide 9
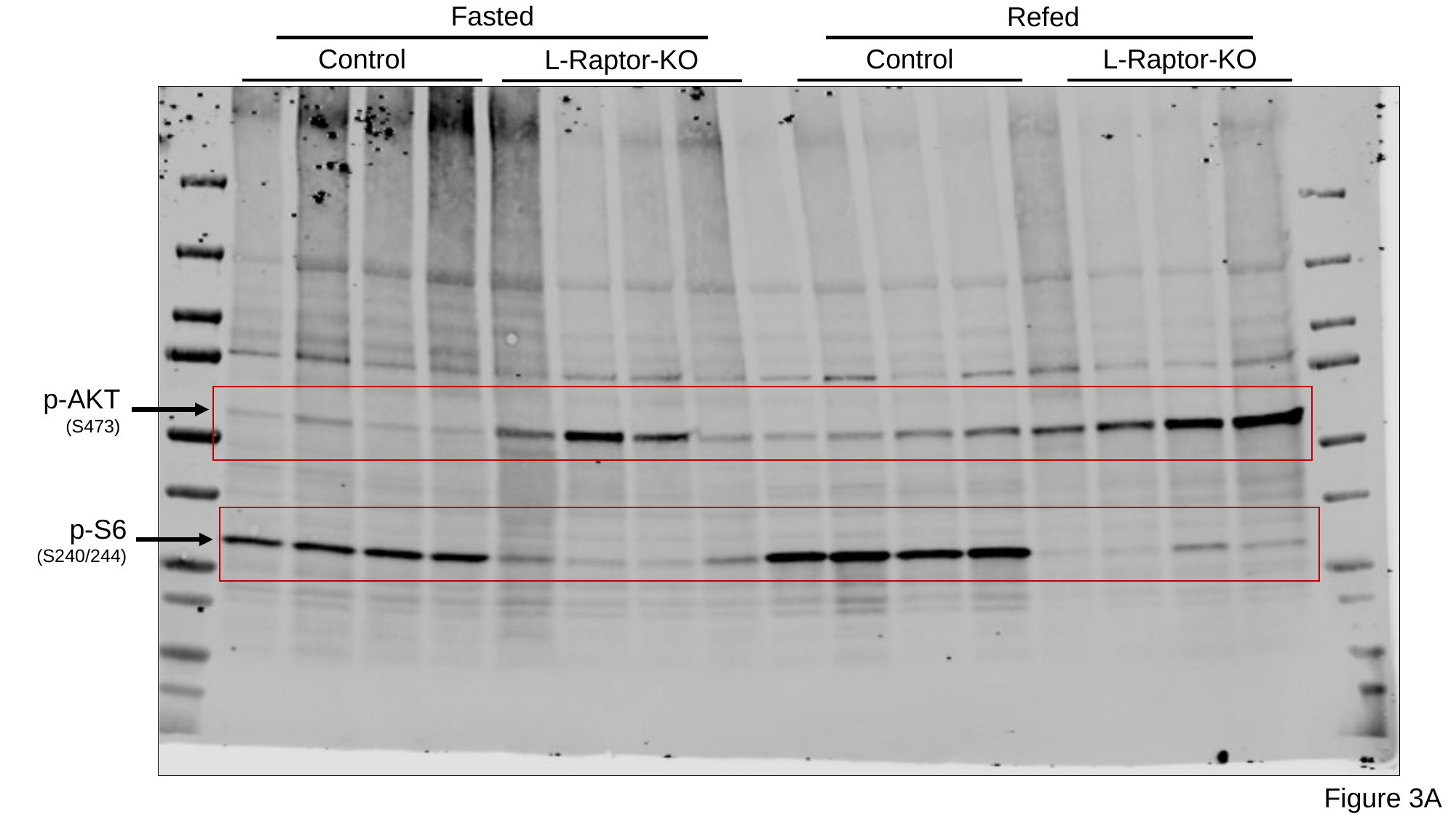

Fasted
Refed
Control
Control
L-Raptor-KO
L-Raptor-KO
p-AKT
(S473)
p-S6
(S240/244)
Figure 3A

## Slide 10
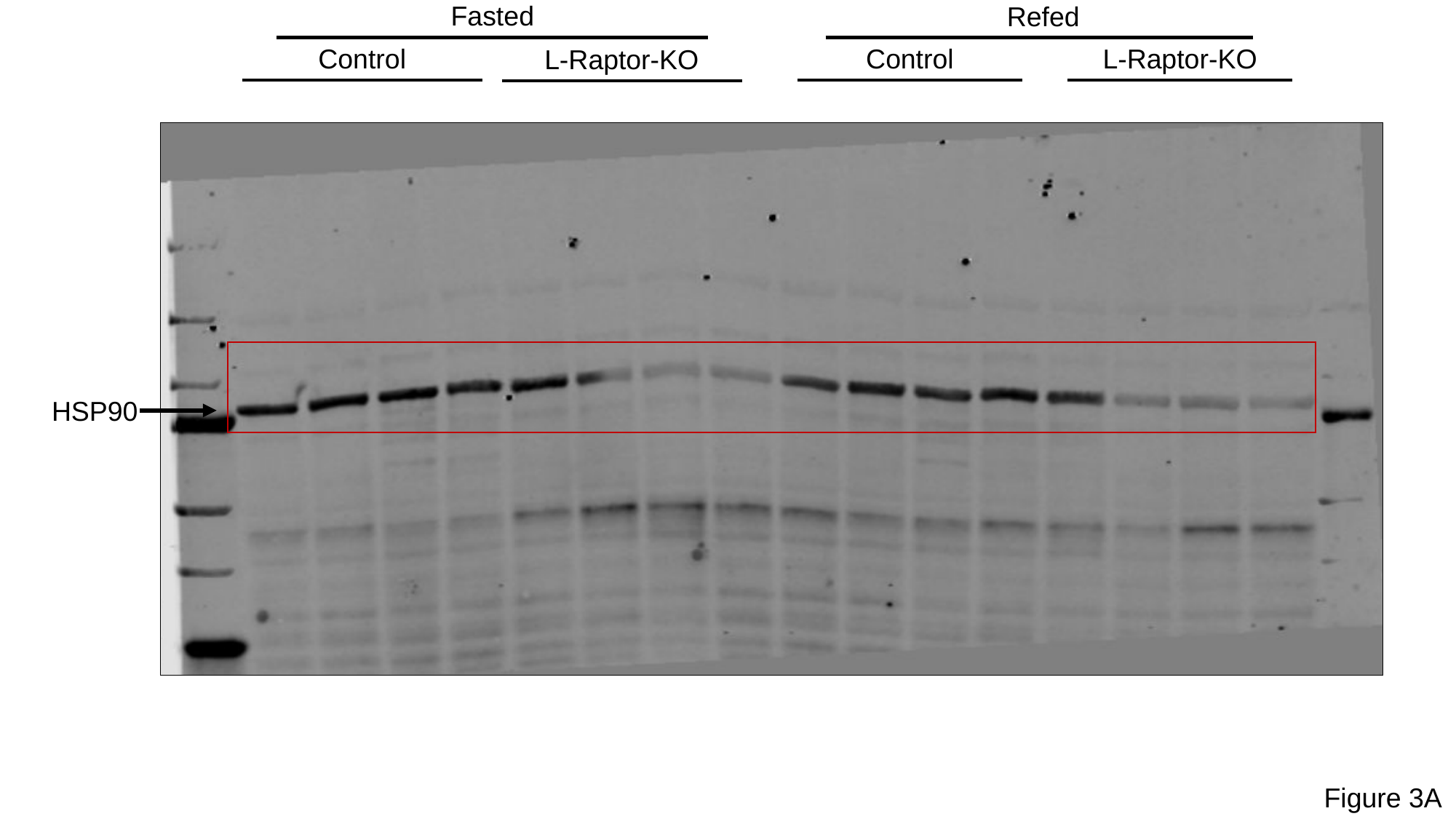

Fasted
Refed
Control
Control
L-Raptor-KO
L-Raptor-KO
HSP90
Figure 3A

## Slide 11
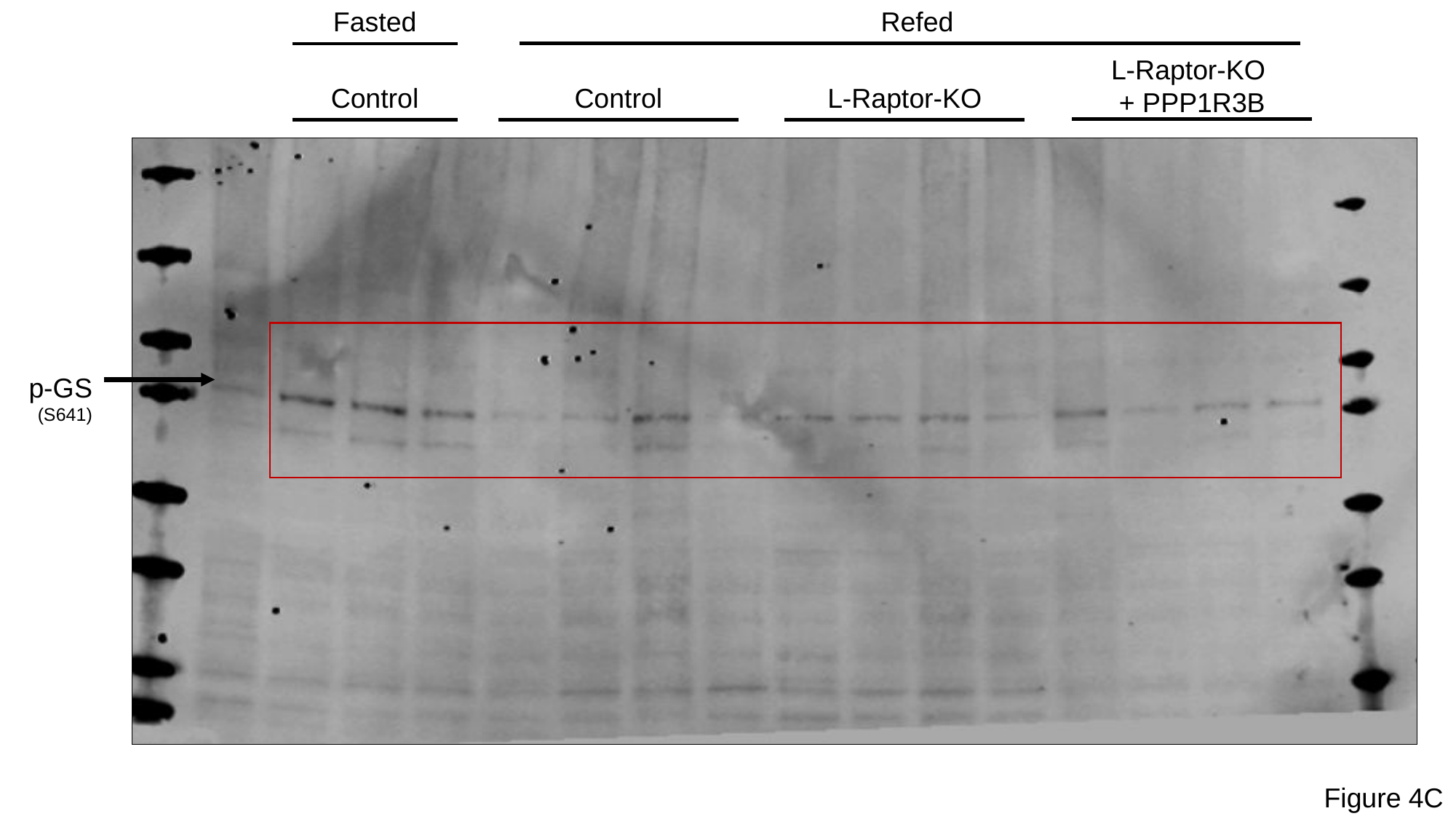

Fasted
Refed
L-Raptor-KO
+ PPP1R3B
Control
Control
L-Raptor-KO
p-GS
(S641)
Figure 4C

## Slide 12
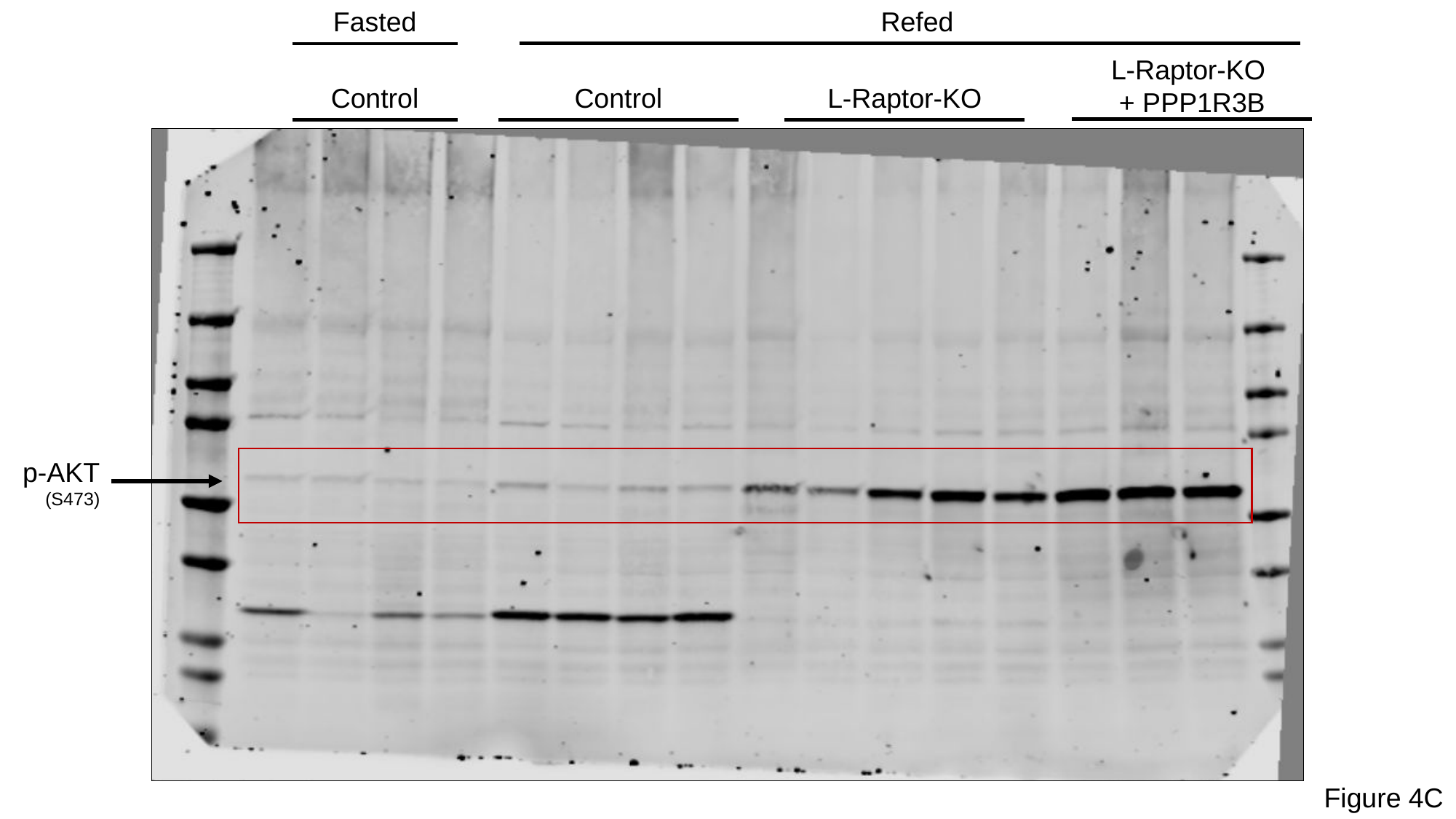

Fasted
Refed
L-Raptor-KO
+ PPP1R3B
Control
Control
L-Raptor-KO
p-AKT
(S473)
Figure 4C

## Slide 13
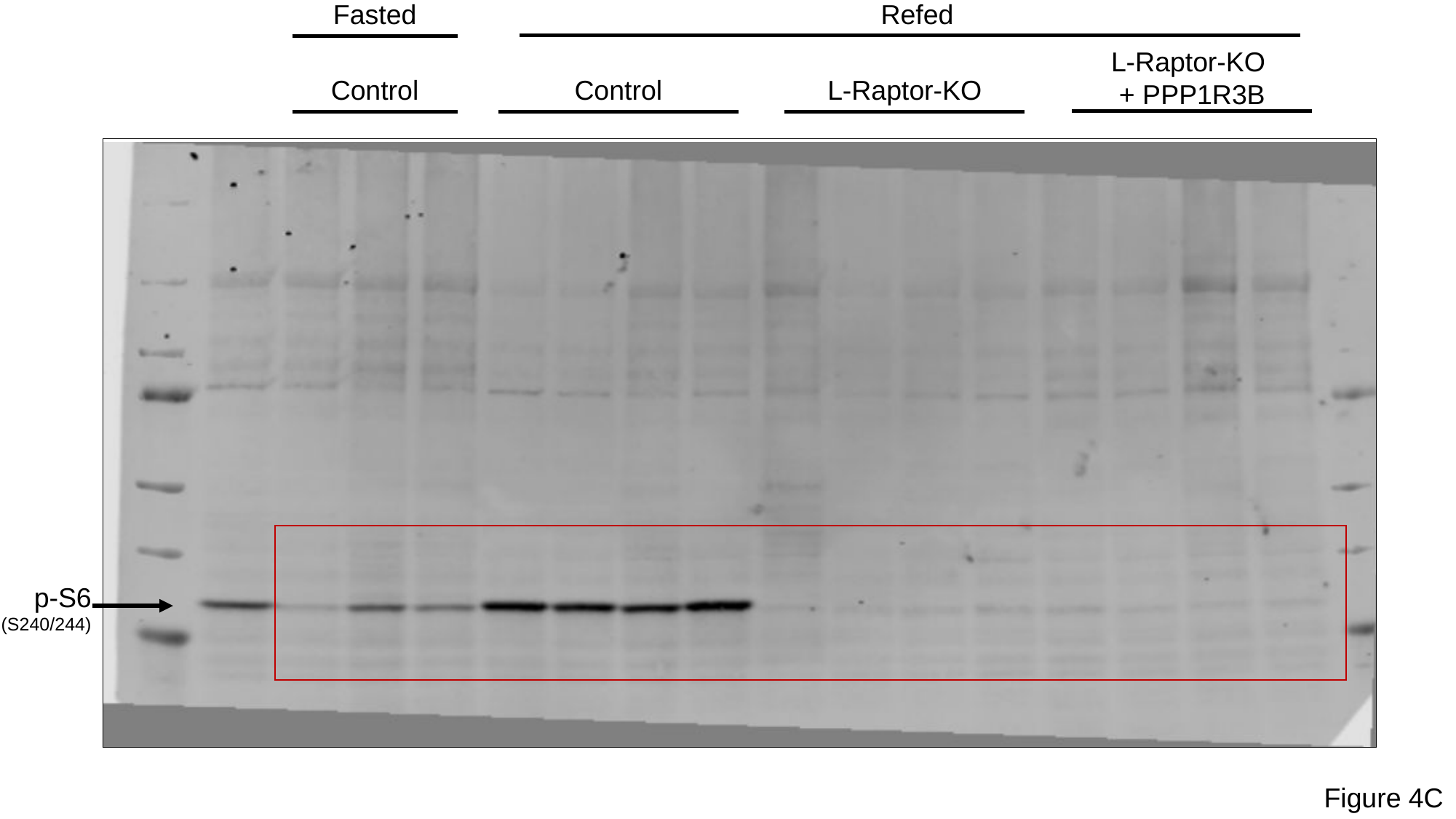

Fasted
Refed
L-Raptor-KO
+ PPP1R3B
Control
Control
L-Raptor-KO
p-S6
(S240/244)
Figure 4C

## Slide 14
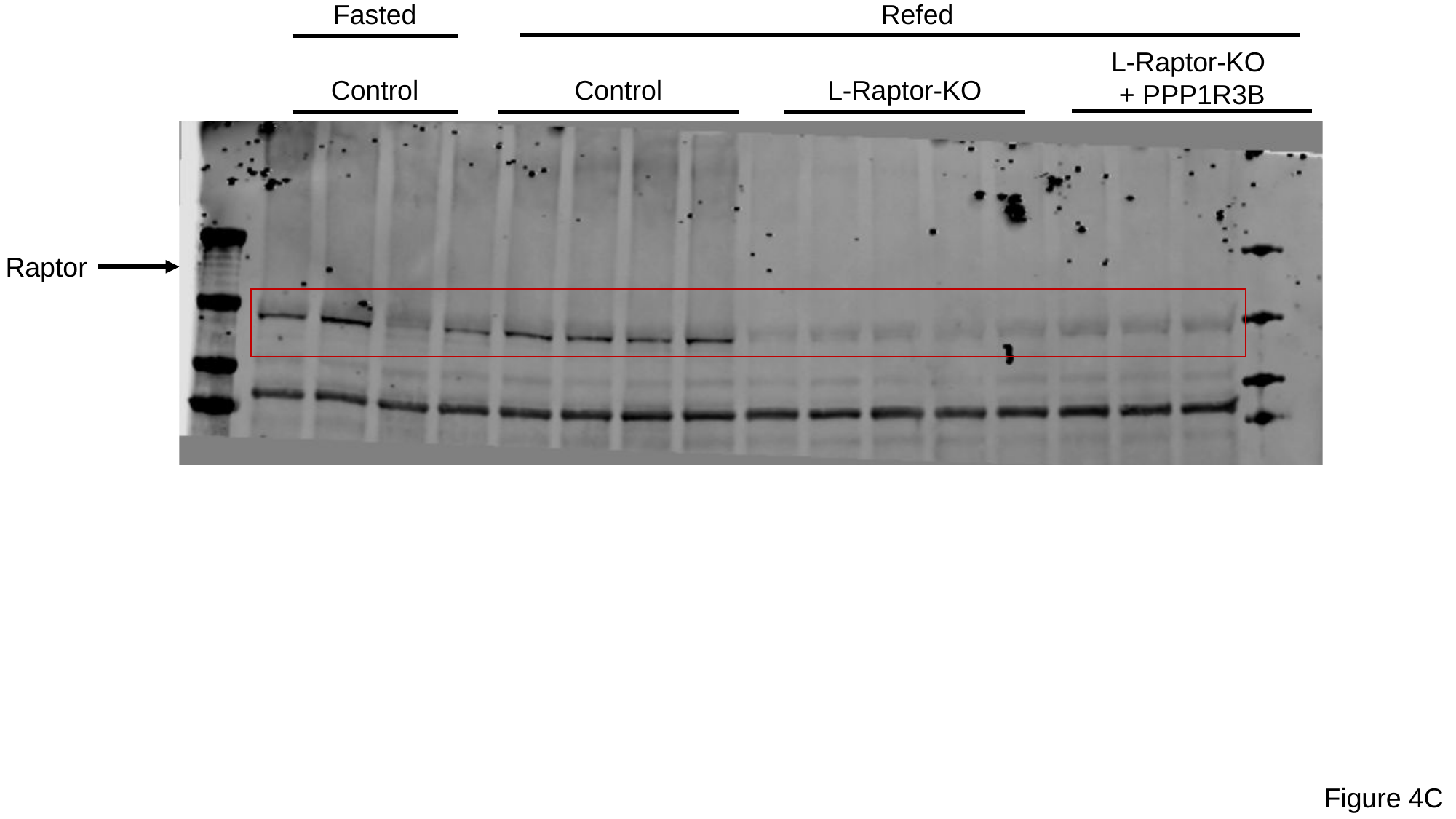

Fasted
Refed
L-Raptor-KO
+ PPP1R3B
Control
Control
L-Raptor-KO
Raptor
Figure 4C

## Slide 15
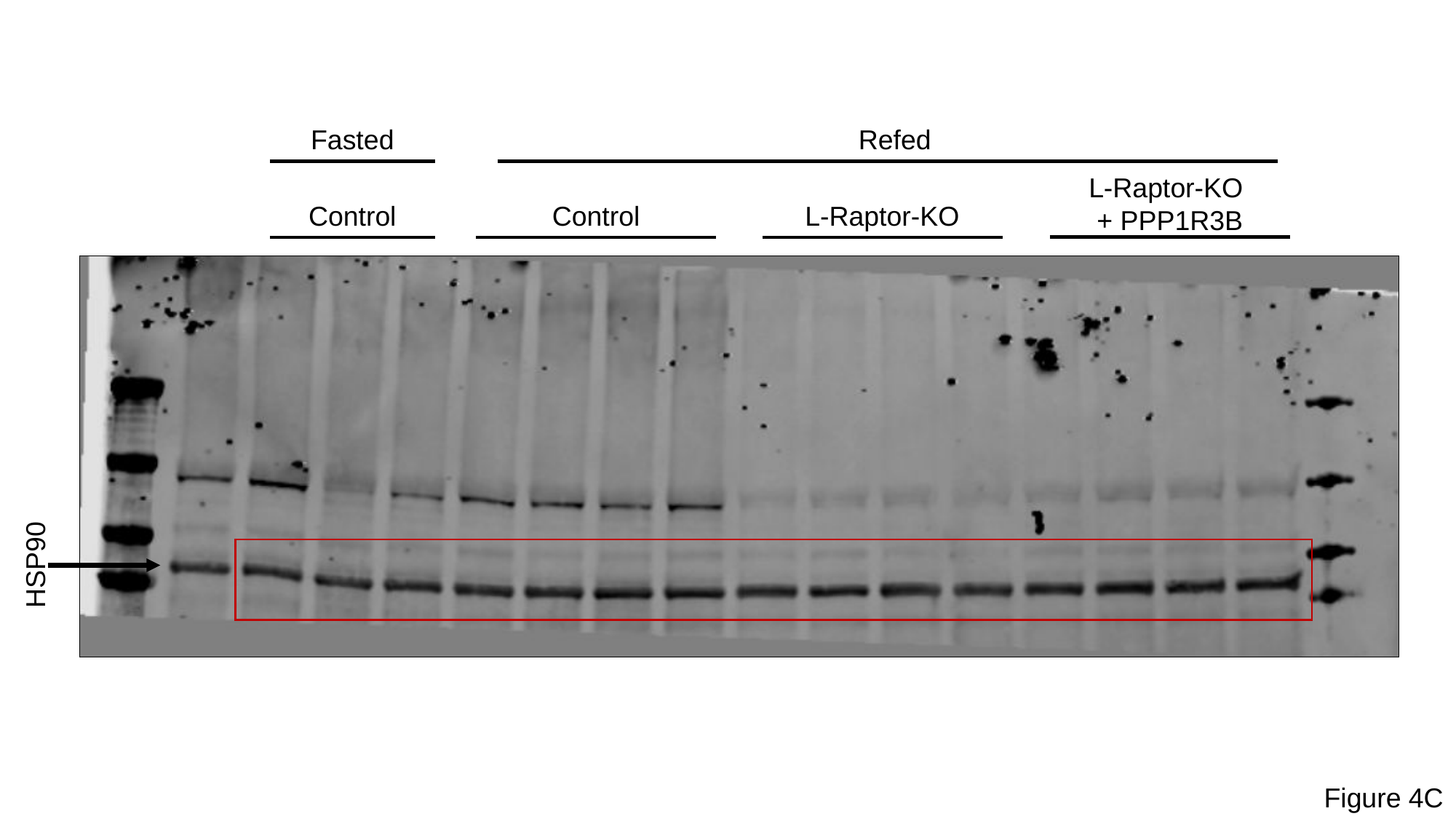

Fasted
Refed
L-Raptor-KO
+ PPP1R3B
Control
Control
L-Raptor-KO
HSP90
Figure 4C

## Slide 16
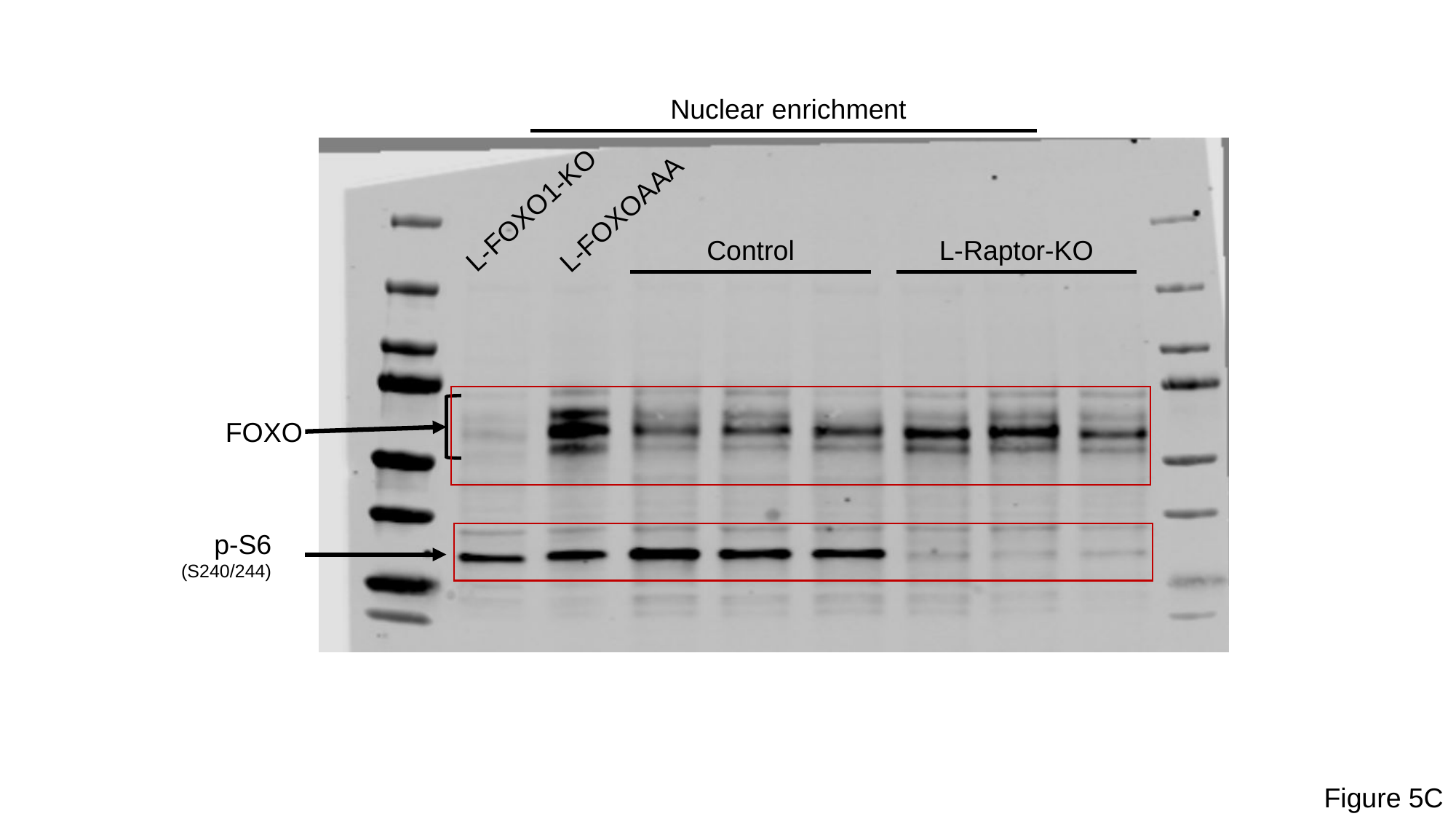

Nuclear enrichment
L-FOXO1-KO
L-FOXOAAA
Control
L-Raptor-KO
FOXO
p-S6
(S240/244)
Figure 5C

## Slide 17
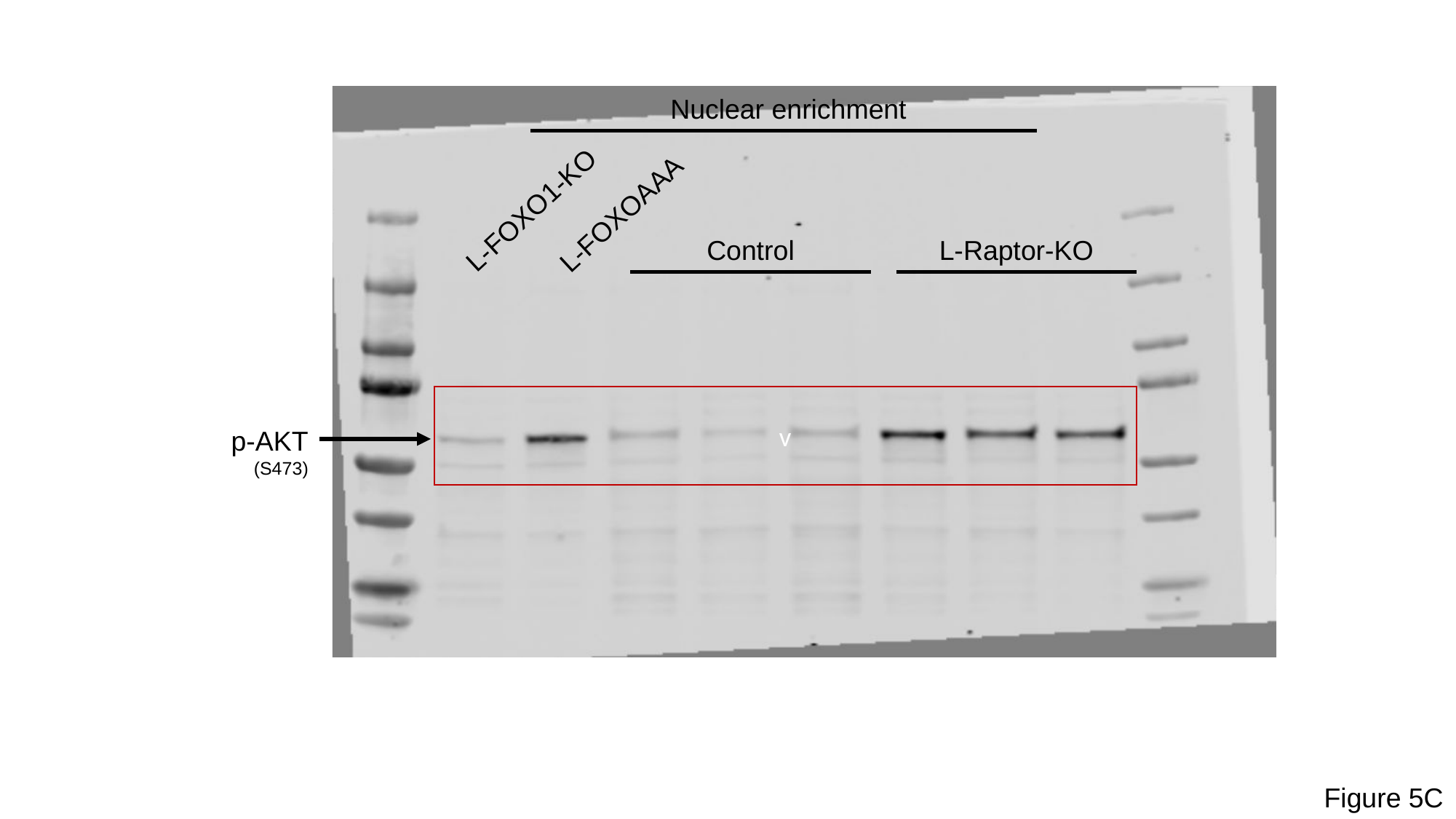

Nuclear enrichment
L-FOXO1-KO
L-FOXOAAA
Control
L-Raptor-KO
v
p-AKT
(S473)
Figure 5C
